# Assessing Machine Learning for Diagnostic Classification of Hypertension Types Identified by Ambulatory Blood Pressure Monitoring

**DOI:** 10.1016/j.cjco.2024.03.005

**Published:** 2024-03-15

**Authors:** Tran Quoc Bao Tran, Stefanie Lip, Clea du Toit, Tejas Kumar Kalaria, Ravi K. Bhaskar, Alison Q. O’Neil, Beata Graff, Michał Hoffmann, Anna Szyndler, Katarzyna Polonis, Jacek Wolf, Sandeep Reddy, Krzysztof Narkiewicz, Indranil Dasgupta, Anna F. Dominiczak, Shyam Visweswaran, Linsay McCallum, Sandosh Padmanabhan

**Affiliations:** a School of Cardiovascular and Metabolic Health, University of Glasgow, Glasgow, United Kingdom; b University Hospitals Birmingham NHS Foundation Trust, Heartlands Hospital, Bordesley Green East, Birmingham, United Kingdom; c Mabgenex, Hyderabad, Telangana, India; d Canon Medical Research Europe, Bonnington Bond, Edinburgh, United Kingdom; e Department of Hypertension and Diabetology, Medical University of Gdańsk, Gdańsk, Poland; f School of Medicine, Deakin University, Geelong, Victoria, Australia; g Department of Biomedical Informatics, University of Pittsburgh, Pittsburgh, Pennsylvania, USA

## Abstract

**Background:**

Inaccurate blood pressure (BP) classification results in inappropriate treatment. We tested whether machine learning (ML), using routine clinical data, can serve as a reliable alternative to ambulatory BP monitoring (ABPM) in classifying BP status.

**Methods:**

This study employed a multicentre approach involving 3 derivation cohorts from Glasgow, Gdańsk, and Birmingham, and a fourth independent evaluation cohort. ML models were trained using office BP, ABPM, and clinical, laboratory, and demographic data, collected from patients referred for hypertension assessment. Seven ML algorithms were trained to classify patients into 5 groups, named as follows: Normal/Target; Hypertension-Masked; Normal/Target-White-Coat (WC); Hypertension-WC; and Hypertension. The 10-year cardiovascular outcomes and 27-year all-cause mortality risks were calculated for the ML-derived groups using the Cox proportional hazards model.

**Results:**

Overall, extreme gradient boosting (using XGBoost open source software) showed the highest area under the receiver operating characteristic curve of 0.85-0.88 across derivation cohorts, Glasgow (n = 923; 43% female; age 50.7 ± 16.3 years), Gdańsk (n = 709; 46% female; age 54.4 ± 13 years), and Birmingham (n = 1222; 56% female; age 55.7 ± 14 years). But accuracy (0.57-0.72) and F1 (harmonic mean of precision and recall) scores (0.57-0.69) were low across the 3 patient cohorts. The evaluation cohort (n = 6213; 51% female; age 51.2 ± 10.8 years) indicated elevated 10-year risks of composite cardiovascular events in the Normal/Target-WC and the Hypertension-WC groups, with heightened 27-year all-cause mortality observed in all groups, except the Hypertension-Masked group, compared to the Normal/Target group.

**Conclusions:**

ML has limited potential in accurate BP classification when ABPM is unavailable. Larger studies including diverse patient groups and different resource settings are warranted.

## Résumé

**Contexte:**

Les erreurs dans la classification des valeurs de la pression artérielle (PA) entraînent une inadéquation du traitement. Nous avons tâché de déterminer si l’apprentissage machine, à l’aide de données cliniques routinières, constituait une solution de rechange fiable à la surveillance ambulatoire de la PA pour définir le statut de la PA.

**Méthodologie:**

Cette étude a utilisé une approche multicentrique incluant trois cohortes de dérivation de Glasgow, Gdańsk et Birmingham, et une quatrième cohorte d’évaluation indépendante. Les modèles d’apprentissage machine ont été développés en analysant les données démographiques, les valeurs de la PA mesurée au cabinet, les données relatives à la surveillance ambulatoire de la PA et aux épreuves de laboratoire recueillies auprès de patients adressés pour une évaluation de l’hypertension. Sept algorithmes d’apprentissage machine ont été appliqués pour classer les patients en cinq groupes : Normale/Cible; Hypertension-Masquée; Normal/Cible-Blouse blanche; Hypertension-Blouse blanche; Hypertension. Les événements cardiovasculaires sur 10 ans et le risque de mortalité toutes causes confondues sur 27 ans ont été calculés dans les groupes dérivés de l’apprentissage machine à l’aide d’un modèle de risques proportionnels de Cox.

**Résultats:**

D’une manière générale, l’amplification de gradient extrême (à l’aide du logiciel ouvert XGBoost) a mis en évidence l’aire sous la courbe de la fonction d’efficacité du récepteur (courbe ROC pour *Receiver Operating Characteristic*) la plus haute, soit 0,85 à 0,88, pour toutes les cohortes de dérivation : Glasgow (n = 923; 43 % de femmes; âge : 50,7 ± 16,3 ans); Gdańsk (n = 709; 46 % de femmes; âge : 54,4 ± 13 ans); Birmingham (n = 1 222; 56 % de femmes; âge : 55,7 ± 14 ans). La précision (0,57 – 0,72) et le score F1 (moyenne harmonique de la précision et du rappel) (0,57 – 0,69) ont été faibles dans les trois cohortes de patients. La cohorte d’évaluation (n = 6 213; 51 % de femmes; âge : 51,2 ± 10,8 ans) a indiqué un risque d’événements cardiovasculaires composites sur 10 ans élevé dans les groupes Normale/Cible-Blouse blanche et Hypertension-Blouse blanche, tandis qu’une hausse de la mortalité toutes causes confondues sur 27 ans a été observée dans tous les groupes, sauf dans le groupe Hypertension-Masquée, comparativement au groupe Normale/Cible.

**Conclusions:**

Le potentiel d’exactitude de la classification de la PA à l’aide de l’apprentissage machine lorsque la surveillance ambulatoire de la PA n’est pas possible est limité. Des études de plus grande envergure portant sur des groupes de patients et des niveaux de ressources diversifiés s’imposent.

Clinical guidelines now recommend out-of-office blood pressure (BP) measurements using ambulatory BP monitoring (ABPM) to screen and diagnose hypertension and monitor on-treatment BP control, as clinic or office BP (oBP) measurement is prone to error.^1^, ^2^, ^3^ Compared to oBP, ABPM is a superior predictor of hypertension-mediated organ damage, and cardiovascular disease (CVD) morbidity and mortality,^3^ and it can identify patients with sustained hypertension, white-coat (WC) hypertension (BP overestimation by oBP measurement), and masked hypertension (BP underestimation by oBP measurement). Those with WC and masked hypertension comprise at least a third of the at-risk population, and masked hypertension is more prevalent in populations of African ancestry.^4^ WC and masked hypertension are associated with an increased risk of progression to sustained hypertension,^5^ highlighting the importance of early identification for more intensive follow-up, even if pharmacologic therapy is not initiated. However, ABPM has several drawbacks, including the limited access to devices (often available only in secondary care); significantly higher device costs compared to oBP measurement devices; staff training; patient discomfort; and sleep disruption.^6^^,^^7^ Any method that can determine true hypertension status without incurring the expense and discomfort of using ABPM will enable effective and timely management in both primary and secondary care. We hypothesized that machine learning (ML) applied to routine clinical data can replace the need for ABPM. Our primary objective was to develop an ML algorithm capable of accurately classifying patients without ABPM measurements into distinct BP risk groups that are as informative as classifications made using ABPM. Our secondary objective was to demonstrate the clinical utility of the ML classification by comparing the risk of CVD morbidity and mortality between these groups. The more-extensive evaluation of all the additional information that ABPM provides (such as BP variability, and circadian variation) was beyond the scope of this study. A validated ML tool that performs well in different settings and is free from algorithmic bias will help hypertension management in both resource-poor and resource-rich settings, allowing clinicians and nurses to effectively manage hypertension by risk-stratifying hypertensive patients, without incurring the added cost or inconvenience to both clinic and patients.

## Methods

### Study datasets

This retrospective study included the following patients: those referred for hypertension assessment to the Glasgow Blood Pressure Clinic^8^ at the Queen Elizabeth University Hospital, Glasgow, United Kingdom (UK) between 2017 and 2019 (Glasgow cohort); patients participating in the CARE NORTH study, a prospective study of hypertensive patients from the outpatient specialist clinic at the Medical University of Gdańsk, Poland (Gdańsk cohort); and patients attending hypertension clinics at Birmingham Heartlands Hospital, Birmingham, UK, between 2001 and 2020 (Birmingham cohort). A temporally distinct cohort for ML-model evaluation was extracted from the Glasgow Blood Pressure Clinic database comprising patients referred to the clinic between 1985 and 2011 and followed-up until 2013 (Glasgow non-ABPM cohort; nonoverlapping with the Glasgow Cohort). Details of the cohorts and ethics statements are provided in Supplemental Appendix S1.

### BP groups

Based on office systolic BP (oSBP) and the average systolic BP from 24-hour ABPM (aSBP), we defined 5 clinically relevant BP groups using BP, oSBP, and aSBP thresholds obtained from current US, European, and UK guidelines.^1^, ^2^, ^3^^,^^9^ These are described in Table 1 and Supplemental Appendix S1.

**Table 1:** Definition of 5 blood pressure (BP) groups

BP group	oSBP	aSBP	oSBP–aSBP
Normal/Target	≤ 140	≤ 135	NA
Hypertension-Masked	≤ 140	> 135	NA
Normal/Target-WC	> 140	≤ 135	NA
Hypertension-WC	> 140	> 135	≥ 15
Hypertension	> 140	> 135	NA

Pressures are given in mm Hg. NA indicates not applicable for that group.

aSBP, ambulatory BP monitoring 24-hour systolic BP average; oSBP, office systolic BP; WC, white-coat.

If both oSBP and aSBP meet nonhypertensive thresholds, this indicates normal SBP in the absence of treatment or at target SBP if on treatment; these patients are grouped together as “Normal/Target.” The group “Hypertension” defines sustained hypertension. The group “Hypertension-Masked” defines hypertensive aSBP in the presence of normal oSBP. Those with a WC effect are divided into 2 groups: those with normal aSBP in the presence of hypertensive oSBP are in the Normal/Target-WC group, whereas those with hypertensive aSBP and oSBP with oSBP ≥ 15 mm Hg higher than aSBP, are in the Hypertension-WC Group. Any difference < 15 mm Hg between the oSBP and the aSBP of an individual with hypertensive oSBP and aSBP qualifies the individual for the Hypertension group. We assigned each patient to 1 of 5 BP groups based on the definitions in Table 1 for the Glasgow, Gdańsk, and Birmingham cohorts. ML models were derived using these group labels.

### Clinical features

Demographics, CVD, antihypertensive therapy, and blood chemistry test results were used to develop the model (see Supplemental Table S1). At the time of ABPM, or within a 3-month window preceding ABPM, features were obtained from standard clinical assessment of patients with hypertension in primary or secondary care who underwent ABPM. However, cohort-specific variations were present in the available features, reflecting differences in clinical practice in the real world. The Glasgow cohort has 14 features; the Gdańsk cohort has 12 features; and the Birmingham cohort has 10 features (see Supplemental Tables S1-S4).

### ML models

All data analysis and ML models were performed in Python 3.8 using Scikit-learn (https://scikit-learn.org/), eXtreme Gradient Boosting (XGBoost open source software, https://github.com/dmlc/xgboost), and Matplotlib software libraries (https://matplotlib.org/). Missing data were imputed using the *k*-nearest neighbor (*k*NN) algorithm. The Shapiro-Wilk test was utilized to examine normality, and the χ^2^ test was utilized to examine the independence of categorical variables.

We derived 7 ML models from clinical features (excluding ABPM, but including oBP measurements), including multinomial logistic regression, support vector machine, *k*NN, naïve Bayes, decision tree, random forest, and tree-based XGBoost.^10^ Using stratified 5-fold cross-validation, separate ML models were developed for each of the 3 cohorts, owing to the distinct features of each and performance metrics reported for each (see Supplemental Appendix S1).

### Performance metrics

Performance of models was reported using the area under the receiver operating characteristic curve (AUROC) and measures calculated from confusion matrices generated for each BP group: accuracy, precision (positive predictive value [PPV]; proportion of relevant instances among the retrieved instances), recall (sensitivity), specificity, F1-score (the harmonic mean of precision and recall), negative predictive value (NPV), and number needed to misdiagnose (NNM; the number of patients who need to be tested in order for 1 to be misdiagnosed by the test). Calibration (the degree of similarity between observed and predicted probability) was assessed by calibration plot and Brier score, as recommended by the Transparent Reporting of a Multivariable Prediction Model for Individual Prognosis or Diagnosis (TRIPOD) guidelines (see Supplemental Appendix S1).^11^ The performance of a model was obtained by averaging the performance across all 5 folds. Results were reported as mean ± standard deviation. In all cases, the significance level was *P* < 0.05.

### Predicting BP group in the Glasgow non-ABPM cohort

Models derived from the Glasgow, Gdańsk, and Birmingham cohorts were used to predict model-specific BP groups for the 7812 patients in the Glasgow non-ABPM cohort. the hypertension duration and the number of antihypertensive medications were not available for the Glasgow non-ABPM cohort, so these features were omitted from the models.

### Survival analysis

Survival analysis was conducted on the Glasgow non-ABPM cohort, by applying models derived from the Glasgow, Gdańsk, and Birmingham ABPM cohorts. The outcomes analyzed were all-cause mortality and CVD composite outcome (defined as time to first CVD admission or CVD death from myocardial infarction, ischemic heart disease, cerebrovascular accident, heart failure, or peripheral vascular disease; see Supplemental Appendix S1). The patients were followed from their first BP clinic visit until death, emigration, or April 1, 2011 (the end of follow-up). Multivariable Cox proportional hazards models were used to assess the prognostic effect of the ML-derived BP groups on all-cause mortality and composite CVD events after adjustment for baseline variables, age, sex, body mass index, cholesterol, smoking status, Charlson comorbidity index, and a variable on year of the first visit strata (epochs) to adjust for secular trends In mortality. Schoenfeld residuals were used to test the proportional hazards assumption. Multiple imputation by chained equations (MICE) was performed for variables with < 10% of values missing (body mass index and cholesterol). Ten imputation datasets were generated, and pooled estimates from Cox regression are reported.

## Results

Table 2 and Supplemental Tables S2-S4 summarize the clinical, laboratory, and demographic characteristics of the 3 study datasets. The proportion of patients on antihypertensive medication varied across cohorts (54% in Glasgow, 96% in Gdańsk, and 42% in Birmingham). The Glasgow cohort had the highest oBP and ABPM readings, and the Gdańsk cohort had the lowest. In Supplemental Tables S2-S4, the distribution of patients across the 5 BP groups, and a summary of the clinical features of the 3 cohorts, are presented. The BP groups with the smallest proportion of patients were the Hypertension-Masked group in the Birmingham cohort (2.1%) and the Glasgow cohort (5.6%), and the Hypertension-WC group in the Gdańsk cohort (6.8%). The Normal/Target-WC group in the Glasgow cohort (37.7%), the Normal/Target-WC group in the Gdańsk cohort (46.3%), and the Normal/Target-WC group in the Birmingham cohort (44.8%) had the highest proportions of patients.

**Table 2:** Summary of patient characteristics

Characteristic	Glasgow	Gdańsk	Birmingham
	n = 923	n = 709	n = 1222
**Demographics**			
Age, y	50.7 ± 16.3	54.4 ± 13.1	55.7 ± 13.9
Sex, % female	43.1	45.6	56.2
**BP measurements & hypertension status**			
oBP, SBP, mm Hg	163.1 ± 22.5	139.9 ± 19.5	157.4 ± 19.9
oBP, DBP, mm Hg	95.4 ± 14.3	82.2 ± 11.2	94.6 ± 12.0
ABPM, SBP, mm Hg	138.0 ± 15.9	129.1 ± 12.3	132.3 ± 13.1
ABPM, DBP, mm Hg	81.5 ± 11.5	77.3 ± 8.9	78.5 ± 9.7
Hypertension duration, y	3.7 ± 4.7	12.1 ± 8.9	—
Prevalent CVD, %	56.6	16.2	7.8
Antihypertensive treatment, % treated	54.0	96.1	42.2
**BP groups**			
Normal/Target	96 (10.4)	328 (46.3)	210 (17.2)
Hypertension-Masked	52 (5.6)	75 (10.6)	26 (2.1)
Normal/Target-WC	348 (37.7)	184 (26.0)	547 (44.8)
Hypertension-WC	249 (27.0)	48 (6.8)	326 (26.7)
Hypertension	178 (19.3)	74 (10.4)	113 (9.2)
**Blood chemistry**			
Sodium, mmol/l	139.4 + 2.5	140.0 ± 2.5	140.1 ± 2.7
Potassium, mmol/l	4.4 ± 0.4	4.2 ± 0.4	4.3 ± 0.4
Creatinine, μmol/l	75.7 ± 19.3	73.6 ± 13.1	80.7 ± 17.7
Cholesterol, mmol/l	5.4 ± 1.2	5.0 ± 1.1	5.4 ± 1.1
Triglyceride, mmol/l	1.9 ± 1.2	1.5 ± 0.8	—
ALT, U/l	28.5 ± 18.6	—	—
Bilirubin, μmol/l	10.5 ± 5.3	—	—

Values are presented as mean ± standard deviation, or proportion (%) of total cohort, unless otherwise indicated. Distribution of patients across 5 blood pressure (BP) groups is also shown. Dash (—) indicates absence of data.

ABPM, ambulatory blood pressure monitoring; ALT, alanine aminotransferase; BP, blood pressure; CVD, cardiovascular disease; DBP, diastolic BP; oBP, office BP; SBP, systolic BP; WC, white-coat.

### Model performance

The performance measures are presented in Table 3 and Supplemental Table S5. Table 3 shows the performance of the XGBoost ML model in classifying patients into distinct BP groups, as defined by ABPM data, which we consider to be the gold standard in this context. Overall, XGBoost and random forest models with AUROC values between 0.85 and 0.88 had the highest level of performance (see Supplemental Figs. S1 and S2). For XGBoost, the accuracy ranged between 0.57 and 0.72, and F1 ranged between 0.57 and 0.69 across the 3 patient cohorts. The simplest *k*NN model (Glasgow and Gdańsk cohorts) and the naïve Bayes model (Glasgow and Birmingham cohorts) performed the worst. The confusion matrices and the corresponding performance metrics for XGBoost classification of patients into the 5 BP groups in the 3 cohorts are depicted in Table 3 and Supplemental Figure S3. Across all 3 cohorts, XGBoost correctly classified 79%-99% of patients in the Normal/Target group, and 60%-80% of patients in the Normal/Target-WC group. The classification accuracies for the Hypertension and the Hypertension-Masked groups were variable across the 3 cohorts, with misclassification into the Normal/Target-WC group and the Normal/Target group, respectively. Supplemental Figure S4 depicts XGBoost calibration.

**Table 3:** Summary of XGBoost performance in the 3 cohorts for each blood pressure (BP) group

Hypertension classification	AUROC	Accuracy	F1 score	Sensitivity	Specificity	PPV
**Glasgow cohort (n = 923)**						
Normal/Target	0.877 ± 0.008	0.574 ± 0.032	0.570 ± 0.032	0.792 ± 0.116	0.971 ± 0.009	0.761 ± 0.048
Hypertension-Masked	0.877 ± 0.008	0.574 ± 0.032	0.570 ± 0.032	0.535 ± 0.182	0.977 ± 0.013	0.587 ± 0.141
Normal/Target-WC	0.877 ± 0.008	0.574 ± 0.032	0.570 ± 0.032	0.604 ± 0.113	0.69 ± 0.014	0.54 ± 0.048
Hypertension-WC	0.877 ± 0.008	0.574 ± 0.032	0.570 ± 0.032	0.582 ± 0.086	0.84 ± 0.045	0.574 ± 0.092
Hypertension	0.877 ± 0.008	0.574 ± 0.032	0.570 ± 0.032	0.399 ± 0.142	0.915 ± 0.031	0.532 ± 0.185
**Gdańsk cohort (n = 709)**						
Normal/Target	0.938 ± 0.004	0.664 ± 0.036	0.729 ± 0.031	0.969 ± 0.051	0.816 ± 0.027	0.82 ± 0.017
Hypertension-Masked	0.938 ± 0.004	0.664 ± 0.036	0.729 ± 0.031	0.067 ± 0.119	0.984 ± 0.026	0.45 ± 0.714
Normal/Target-WC	0.938 ± 0.004	0.664 ± 0.036	0.729 ± 0.031	0.913 ± 0.054	0.819 ± 0.028	0.639 ± 0.038
Hypertension-WC	0.938 ± 0.004	0.664 ± 0.036	0.729 ± 0.031	0.371 ± 0.466	0.983 ± 0.02	0.654 ± 0.523
Hypertension	0.938 ± 0.004	0.664 ± 0.036	0.729 ± 0.031	0.107 ± 0.16	0.991 ± 0.025	0.69 ± 0.623
**Birmingham cohort (n = 1222)**						
Normal/Target	0.937 ± 0.010	0.692 ± 0.047	0.724 ± 0.045	0.986 ± 0.038	0.974 ± 0.004	0.888 ± 0.014
Hypertension-Masked	0.937 ± 0.010	0.692 ± 0.047	0.724 ± 0.045	0.0 ± 0.0	0.997 ± 0.007	0.0 ± 0.0
Normal/Target-WC	0.937 ± 0.010	0.692 ± 0.047	0.724 ± 0.045	0.812 ± 0.081	0.716 ± 0.085	0.699 ± 0.09
Hypertension-WC	0.937 ± 0.010	0.692 ± 0.047	0.724 ± 0.045	0.687 ± 0.134	0.884 ± 0.038	0.684 ± 0.069
Hypertension	0.937 ± 0.010	0.692 ± 0.047	0.724 ± 0.045	0.089 ± 0.101	0.989 ± 0.016	0.521 ± 0.538

Sensitivity, specificity, and positive predictive value (precision; PPV) are reported for each fold. The F1 score is the harmonic mean of precision and recall.

AUROC, area under the receiver operating characteristic curve; WC, white coat.; XGBoost, eXtreme Gradient Boosting open source software.

### Feature importance

The XGBoost model revealed that in all cohorts, oSBP, age, cholesterol, and creatinine level were the most influential predictors (see Supplemental Fig. S5). Substantial variations were present in influential characteristics among models and among cohorts (see Supplemental Fig. S6). XGBoost demonstrated a balanced contribution from all features, whereas other models exhibited a predominance of 1 to 3 features in determining model performance (Supplemental Fig. S6).

### Survival analysis

The demographic characteristics of the Glasgow non-ABPM cohort are summarized in Supplemental Table S6. The results of Cox regression analysis are displayed in Table 4 and Supplemental Figure S7. A significantly higher 10-year risk of composite CVD events was evident for the Normal/Target-WC group (hazard ratio (HR) 1.3 [95% CI: 1.09; 1.55], *P* = 0.003) and the Hypertension-WC group (HR 1.38 [95% CI: 1.14; 1.67], *P* = 0.001), compared to that for the Normal/Target group when predicted by the model derived from the Glasgow cohort. When heterologous models—that is, Birmingham or Gdańsk XGBoost models—were applied to the Glasgow non-ABPM cohort, the outcomes were largely consistent. Hypertension-WC groups predicted by the Birmingham (HR 1.42 [95% CI: 1.21; 1.66], *P* < 0.0001) and Gdańsk (HR 1.33 [95% CI: 1.12; 1.57], *P* = 0.001) XGBoost models were associated with a higher 10-year CVD risk, compared to that for the Normal/Target group. The predicted Normal-Target WC groups did not show an increased risk of 10-year cardiovascular events for the Birmingham cohort (HR 1.16 [95% CI: 0.99; 1.36], *P* = 0.059) or the Gdańsk cohort (HR 1.16 [95% CI: 0.96; 1.39], *P* = 0.127). An interesting finding is that the Glasgow and Gdańsk models did not reveal an increased risk of CVD outcomes in the Hypertension group. The Normal/Target-WC and the Hypertension-WC groups showed significantly higher all-cause mortality compared to the Normal/Target group for all 3 models. The Hypertension group showed significantly increased mortality risk with the Glasgow and Gdańsk models. The Hypertension-Masked group showed significantly increased mortality risk with only the Glasgow model. For all-cause mortality outcomes, the Normal/Target-WC group (Glasgow: HR 1.42 [95% CI: 1.17; 1.73], *P* = 0.0004; Birmingham: HR 1.2 [95% CI: 1.01; 1.42], *P* = 0.036; Gdańsk: HR 1.25 [1.03; 1.52], *P* = 0.025) and the Hypertension-WC group (Glasgow:HR 1.69 [95% CI: 1.38; 2.07], *P* < 0.0001; Birmingham: HR 1.5 [95% CI: 1.27; 1.76], *P* < 0.0001; Gdańsk: HR1.39 [95% CI: 1.17; 1.66], *P* = 0.0002) showed significant associations regardless of the classification model used. All-cause mortality for the Hypertension group did not attain statistical significance for the Birmingham model (Glasgow: HR 1.33 [95% CI: 1.02; 1.74], *P* = 0.034; Birmingham: HR 1.44 [95% CI: 0.99; 2.07], *P* = 0.052; Gdansk: HR 1.23 [95% CI: 1.0;1.51], *P* = 0.049), and the Hypertension-Masked group showed an increased risk of death with only the Glasgow model (HR 1.35 [95% CI: 1.01; 1.82], *P* = 0.046).

**Table 4:** Results of Cox regression analysis for 27-year all-cause mortality and for 10-year composite cardiovascular events

Analysis	XGBoost model		
	Glasgow cohort	Birmingham cohort	Gdańsk cohort
**27-y all-cause mortality**			
Normal/Target	1	1	1
Normal/Target-WC	1.42 [1.17; 1.73] *P*= 0.0004	1.2 [1.01; 1.42] *P* = 0.036	1.25 [1.03; 1.52] *P* = 0.025
Hypertension-Masked	1.35 [1.01; 1.82] *P* = 0.046	0.4 [0.06; 2.86] *P* = 0.362	0.85 [0.59; 1.22] *P* = 0.365
Hypertension-WC	1.69 [1.38; 2.07] *P* < 0.0001	1.5 [1.27; 1.76] *P* < 0.0001	1.39 [1.17; 1.66] *P* = 0.0002
Hypertension	1.33 [1.02; 1.74] *P* = 0.034	1.44 [0.99; 2.07] *P* = 0.052	1.23 [1.0; 1.51] *P* = 0.049
**10-y composite cardiovascular events**			
Normal/Target	1	1	1
Normal/Target-WC	1.3 [1.09; 1.55] *P* = 0.003	1.16 [0.99; 1.36] *P* = 0.059	1.16 [0.96; 1.39] *P* = 0.127
Hypertension-Masked	1.03 [0.77; 1.37] *P* = 0.853	1.47 [0.47; 4.6] *P* = 0.509	0.83 [0.60; 1.14] *P* = 0.24
Hypertension-WC	1.38 [1.14; 1.67] *P* = 0.001	1.42 [1.21; 1.66] *P* < 0.0001	1.33 [1.12; 1.57] *P* = 0.001
Hypertension	1.05 [0.81; 1.35] *P* = 0.708	1.4 [0.99; 1.98] *P* = 0.058	1.13 [0.93; 1.37] *P* = 0.223

The values represent adjusted hazard ratios and 95% confidence intervals.

WC, white coat; XGBoost, eXtreme Gradient Boosting open source software.

## Discussion

In our proof-of-concept investigation, we explored the viability of using ML applied to routine clinical data as a substitute for ABPM in the clinical management of hypertension. Despite the modest accuracy of the ML classifications, our analysis of associations with clinical outcomes revealed that certain ML-derived categories correlate with a higher risk, compared to that for the reference category of normal/target BP. This observation suggests that even with current accuracy limitations, ML classifications can provide preliminary insights into risk stratification that merit further investigation. The implications for our results, despite the acknowledged low accuracy levels, are 2-fold. First, the study demonstrates the potential clinical relevance of ML classifications in identifying risk groups, which could be refined and validated in larger, more diverse datasets. Second, the study underscores the importance of enhancing accuracy and reliability in future research. The observed associations, despite the current limitations, indicate a promising direction for leveraging ML in hypertension management, especially in contexts in which ABPM is not feasible. An interesting insight from our study is the potential generalizability of the ML model, regardless of the originating cohort, in consistently identifying individuals exhibiting the WC effect, with consequent increased cardiovascular risk. Our approach to classifying individuals into 5 risk groups differs from previous efforts, which focused primarily on deducing ABPM BP values from oBP measurements and other clinical parameters.^12^

Our ML algorithms exhibited some degree of misclassification across all 3 cohorts, particularly between the Normal/Target and the Hypertension-Masked groups, and within the trio of the Normal/Target-WC, the Hypertension-WC, and the Hypertension groups. Although the models showed high AUROCs, these misclassifications resulted in suboptimal calibration metrics.

The XGBoost model performed better in the Glasgow cohort (40%) in the detection of the Hypertension and Hypertension-Masked groups (54%), compared to the Gdańsk and Birmingham cohorts (20% and 9% for the Hypertension group, and 20% and 0% for the Hypertension-Masked group, respectively). The relatively higher detection rate for the smaller Hypertension-Masked and Hypertension groups in the Glasgow cohort may be attributable to the Glasgow cohort having more input features (Supplemental Table S1) and a more balanced distribution of groups between the 2 cohorts (Supplemental Tables S2-S4). The advantage of XGBoost models is that they harmonize a range of covariates, including demographic and lab values, to construct the prediction, with no single covariate predominating, in contrast to logistic regression (see Supplemental Fig. S6). Nevertheless, the development of ML models from unbalanced data can result in predictions that are skewed toward the majority group and toward deceptively high performance. Typically, this phenomenon is referred to as the accuracy paradox. Consequently, despite the Glasgow cohor’'s superior predictive ability for smaller subgroups, its model produced a lower level of accuracy, and lower F1 and AUROC scores than those of the Gdańsk and Birmingham cohorts. Performance matrices (Supplemental Table S5), which provide a more in-depth analysis of the mode’'s classification accuracy, demonstrated that the Glasgow cohor’'s model has the best performance in detecting clinically significant smaller subgroups. Oversampling implicitly increases the prior probability of smaller subgroups, which could lead to misclassifications of patients from the majority group.

ABPM is considered the gold standard for diagnosing hypertension.^2^^,^^3^^,^^9^^,^^13^ ABPM provides a more precise hypertension diagnosis, which benefits both the individual patient and the healthcare system. Avoiding unnecessary antihypertensive treatment in patients has resulted in a 3%-14% decrease in treatment costs. However, due to the requirements for technology and other resources, the immediate cost of ABPM is significantly higher than that of routine clinic BP measurement.^6^^,^^7^ ABPM is also more taxing on patients, as the BP cuff inflates and deflates at least twice or thrice hourly: one third of patients report having pain or bruising during the 24-hour monitoring process, and two thirds report having sleep disturbances.

The primary strengths of our study are the following: the development of multiple ML models in 3 temporally and geographically distinct cohorts, which strengthen the stud’'s conclusions; the use of cross-validation to generate robust confidence intervals for our estimates; the demonstration of an impact on long-term outcomes; and adherence to the current Transparent Reporting of a Multivariable Prediction Model for Individual Prognosis or Diagnosis-Artificial Intelligence (TRIPOD-AI) guidelines for reporting prediction models.^11^ However, our study did not come without limitations. In addition to the misclassification and poor calibration metrics noted above, some of the BP groups were underrepresented due to the small sample size, most notably the Hypertension-Masked group, and the majority of patients were White Europeans with little ethnic diversity. We were unable to account for secular effects among cohorts in the survival analyses, and we used only the year of inclusion as a covariate to address this issue. The Glasgow non-ABPM cohort comprised hypertensive patients attending the Glasgow BP clinic—thus, all were managed nearly similarly at a specialist clinic. The discrepancy in the cardiovascular outcomes risk seen in the Hypertensive group and the WC group, compared to that in the Normal/Target BP group, on applying models derived from the 3 cohorts on outcomes in the Glasgow non-ABPM cohort, may reflect an existing unmet need, with those patients with the WC effect still being undertreated. We surmise that further research using larger datasets will help determine the predictive power of the ML models and clarify the implications for long-term outcomes.

This study serves as a foundational proof-of-concept, demonstrating the potential of ML to categorize patients into clinically pertinent risk groups when ABPM is unavailable, although it underscores that ML cannot supplant ABPM completely at this juncture. Our findings advocate for the conduct of more extensive prospective studies, with the inclusion of a diverse array of racial and ethnic groups, and a variety of resource settings, alongside adequate representation across all BP classifications. Furthermore, the successful and ethical deployment of ML in healthcare, particularly in resource-poor settings, requires a participatory approach that includes patients, healthcare providers, and other stakeholders from these communities from the outset. In conclusion, a comprehensive evaluation is essential to determine the true potential of ML as either a direct substitute for or a significant complement to ABPM. By integrating ML into the clinical pathway, we have the opportunity to revolutionize the management of hypertension, enabling timely and effective interventions across diverse healthcare environments.
