# Supplementary material for: Assessing Machine Learning for Diagnostic Classification of Hypertension Types Identified by Ambulatory Blood Pressure Monitoring
Source: CJC Open. 2024 Mar 15;6(6):798–804. doi: 10.1016/j.cjco.2024.03.005 (PMC11250845; doi:10.1016/j.cjco.2024.03.005)
Supplement: Supplementary Material [file mmc1.docx]

Supplementary Appendix

Predictive accuracy and clinical utility of machine learning prediction for classifying blood pressure status

Tran Quoc Bao Tran MSc,*^1^ Stefanie Lip MBChB,*^1^ Clea du Toit MSc,*^1^ Tejas Kumar Kalaria MRCP,^2^ Ravi K Bhaskar,^3^ Alison Q O’Neil EngD,^4^ Beata Graff MD PhD,^5^ Michał Hoffmann MD PhD,^5^ Anna Szyndler MD PhD,^5^ Katarzyna Polonis PhD,^5^ Jacek Wolf MD PhD,^5^ Sandeep Reddy MBBS PhD,^6^ Krzysztof Narkiewicz MD PhD,^5^ Indranil Dasgupta DM,^2,7^ Anna F. Dominiczak MD FMedSci,^1^ Shyam Visweswaran MD PhD,^8^ Linsay McCallum PhD,^1^ Sandosh Padmanabhan^1^ MD PhD

Contents

[Supplementary Methods 3](#_Toc108167385)

[Patient Cohorts 3](#_Toc108167386)

[Cross-validation 4](#_Toc108167387)

[Hyperparameter optimisation 4](#_Toc108167388)

[Calibration curve 5](#_Toc108167389)

[Evaluation Criteria 5](#_Toc108167390)

[Feature Importance 6](#_Toc108167391)

[Supplementary Figures 7](#_Toc108167392)

[Supplementary Figure 1: 7](#_Toc108167393)

[Supplementary Figure 2: 8](#_Toc108167394)

[Supplementary Figure 3: 11](#_Toc108167395)

[Supplementary Figure 4: 12](#_Toc108167396)

[Supplementary Figure 5: 15](#_Toc108167397)

[Supplementary Figure 6: 16](#_Toc108167398)

[Supplementary Figure 7: 19](#_Toc108167399)

[Supplementary Tables 20](#_Toc108167400)

[Supplementary Table 1: 20](#_Toc108167401)

[Supplementary Table 2: 21](#_Toc108167402)

[Supplementary Table 3: 22](#_Toc108167403)

[Supplementary Table 4: 23](#_Toc108167404)

[Supplementary Table 5: 23](#_Toc108167405)

[Supplementary Table 6: 25](#_Toc108167406)

[Supplementary Table 7: 27](#_Toc108167407)

# Supplementary Methods

## Patient Cohorts

**Glasgow Cohort**

ABPM and oBP measurements, clinical parameters, and blood chemistry results were collected from 926 patients attending the Glasgow Blood Pressure Clinic (GBPC) at the Queen Elizabeth University Hospital (QEUH) between 2017-2019. The GBPC, located in the Greater Glasgow area, is the main specialist hypertension clinic in Glasgow, providing secondary- and tertiary-level services to patients with hypertension in the West of Scotland. At each visit, BP measurements were taken 3 times by a specialist hypertension nurse using a validated digital OMRON monitor; the mean of the last 2 measurements was recorded. Patients attending the clinic were advised to take their regular medications before the clinic visit. The specialist nurse confirmed medical history and drug history at the time of BP recording. Blood samples were taken as part of usual clinical care and processed in the NHS Greater Glasgow and Clyde (NHSGGC) laboratory using standard techniques. The ABPM (Spacelabs 90217RM) was performed as part of usual clinical care and BP was measured for 24 hours with daytime readings every 30 minutes (0800–2159) and night-time readings every 60 minutes (2200–0759).

Further details on the study population and measurements have been described previously.^1^ In brief, patients were referred to the GBPC if their BP was not controlled in primary care with at least 3 drugs or if there was evidence of high-risk factors such as early-onset hypertension and a family history of premature CVD or concern over secondary causes. Structured data collection forms were used to collect information from all patients attending the clinic, and the data were stored electronically in a single computerised database.

**Glasgow Non-ABPM Cohort**

A temporally distinct cohort, non-overlapping with the Glasgow Cohort was extracted from the GBPC database; this cohort comprised 5,092 patients aged between 30 and 70 years referred to the clinic between 1985 and 2011 and followed up until 2013. All patients had oBP recorded but ABPM data were not available. Hospital admissions and mortality in the Glasgow non-ABPM cohort were available from 1985 to March 2011 and were obtained from the NHS Information and Statistics Division. In Scotland, the NHS provides primary and secondary health care to all citizens, free at the point of access. The diagnoses from the patients’ admissions were available from the Information and Statistics Division, coded according to the World Health Organization Classification of Diseases (International Classification of Diseases–Ninth Revision before 1996 and International Classification of Diseases–Tenth Revision after 1996). Comorbidities at baseline and 1-year were determined using the Charlson comorbidity score.

**Gdańsk Cohort**

Both ABPM and oBP measurements were collected from patients participating in the CARE NORTH study which was a prospective study of hypertensive patients from the outpatient specialist clinic at the Medical University of Gdańsk, Poland. The patients (n=854) were consecutively recruited into the study between 2009 and 2011 after giving informed consent, and a subset of 709 patients was chosen for further analysis based on the availability of data. The complete baseline examination (a medical history review, physical examination, arterial stiffness assessment, ABPM, and blood and urinary samples collection) was performed by medical staff during scheduled visits. BP measurements were taken 3 times on the non-dominant arm, and the average of 3 readings was taken for further analysis. ABPM was measured with SpaceLabs 90207 and SpaceLabs 90217 devices set at 20 min daytime and 30 min night-time intervals.

**Birmingham Cohort**

Data were collected from all patients attending Birmingham Heartlands Hospital hypertension clinics between 2001 and 2020 and recorded in a dedicated database. These patients were representative of a typical secondary care hypertension clinic population in the UK. The indications for a referral from primary care were resistant hypertension, multiple drug intolerances, investigation of secondary hypertension or suspected white coat hypertension. Patients had oBP and ABPM recorded at their initial appointment in addition to age, sex, self-reported ethnicity, height, weight, smoking status, diabetes status, and serum chemistry. The database was anonymised for analysis; patients with incomplete data were excluded. The oBP was measured by trained nurses using validated automated devices (Omron M5-I). Clinic protocol involved measuring seated BP from one or both upper arms following a period of 5 minutes rest. Three oBP readings were taken for each patient, and the average taken as the clinic reading. Ambulatory BP monitors were fitted on the same day as clinic readings. ABPM measurement was performed using SpaceLabs 90207 and 90217; daytime measurements were recorded from 07:00 to 23:00 every 30 minutes and night-time measurements were recorded every 60 minutes.

## Ethics statements

Use of the GBPC data was approved by the London - Chelsea Research Ethics Committee (20/PR/0229; protocol 1-250620) and NHSGGC Research and Innovation (GN20CA267). The West of Scotland Research Ethics Service of the National Health Service (NHS) approved the analysis of anonymised data from the GBPC database (11/WS/0083), the source of the Glasgow non-ABPM cohort. The Ethical Committee of the University of Gdańsk, Poland approved the CARE NORTH study (NKEBN/285/2009). The Birmingham data were collected as part of a service improvement project and were anonymized for research. The study did not require Research Ethics Committee approval (Personal communication, Health Research Authority, UK; 27 April 2021; [queries@hra.nhs.uk](mailto:queries@hra.nhs.uk).)

## BP Groups Classification

If both oSBP and aSBP meet non-hypertensive thresholds, this indicates normal SBP in the absence of treatment or at target SBP if on treatment; these patients are grouped together as ‘Normal/Target’. The group ‘Hypertension’ defines sustained hypertension. The group ‘Hypertension-Masked’ defines hypertensive aSBP in the presence of normal oSBP. Those with a WC effect are divided into two groups: normal aSBP in the presence of hypertensive oSBP are labelled ‘Normal/Target-WC’, whereas those with hypertensive aSBP and oSBP where oSBP is >=15 mmHg higher than aSBP are labelled ‘Hypertension-WC’. Any difference <15mmHg between the oSBP and aSBP of an individual with hypertensive oSBP and aSBP qualifies the individual for the group ‘Hypertension’. We assigned each patient to one of five BP groups based on the definitions in **Table 1** for the Glasgow, Gdańsk, and Birmingham cohorts. ML models were derived using these labels.

## Survival Analysis

The patients were followed from their first BP clinic visit until death, emigration, or April 1, 2011 (the end of follow-up). Multivariable Cox proportional hazards models were used to assess the prognostic effect of the ML-derived BP groups on all-cause mortality and composite CVD events after adjustment for baseline variables, age, sex, body mass index (BMI), cholesterol, smoking status, Charlson comorbidity index, and a variable on year of the first visit strata (epochs) to adjust for secular trends in mortality. Schoenfeld residuals were used to test the proportional hazards assumption. Multiple imputation by chained equations (MICE) was performed for variables with <10% of values missing (BMI and cholesterol). Ten imputation datasets were generated, and pooled estimates from Cox regression are reported.

## Cross-validation

First, the dataset was partitioned into five equal sections, or folds, such that the proportion of the BP groups is the same in each fold as it is in the full dataset. During the training process, four folds (80% of the dataset) were used for training and one fold (the remaining 20% of the dataset) was used for testing.^2-4^ This process was repeated five times, so that each fold was used once as the test set. The study design used for an example ML algorithm is illustrated in Figure 1.

## Performance Metrics

Performance of models was reported using the area under the receiver operating characteristic curve (AUROC) and measures calculated from confusion matrices generated for each BP group: accuracy, precision (positive predictive value (PPV); proportion of relevant instances among the retrieved instances), recall (sensitivity), specificity, F1-score (the harmonic mean of precision and recall), negative predictive value (NPV), and number needed to misdiagnose (the number of patients who need to be tested in order for one to be misdiagnosed by the test; NNM). Calibration (the degree of similarity between observed and predicted probability) was assessed by calibration plot and Brier score as recommended by TRIPOD guidelines.^5^ The performance of a model was obtained by averaging the performance across all five folds. Results were reported as mean ± standard deviation (SD). In all cases, the significance level was p<0.05.

## Hyperparameter optimisation

Some of the ML algorithms employ hyperparameters, which are a set of parameters that control the design of the model architecture and training process. To determine the optimal values of the hyperparameters for each model, a grid search was applied separately to each of the five folds to maximise the F1 score.^3, 4^

For tree-based models (DT, RF, XGB), hyperparameter optimization was carried out using nested cross-validation, in which two cross-validation loops were performed in parallel: scikit-learn’s GridsearchCV forms the inner loop to evaluate the hyperparameters, while sklearn’s cross_val_score forms the outer loop to measure the prediction performance of the estimator based on the selected hyperparameters.^6^ The resulting scores were thus the unbiased estimates of the prediction score on the validation data.^7^ Weighted F1 score was used as the scoring criterion for GridsearchCV. For the NB model, instead of hyperparameters, different types of Naïve Bayes algorithms (Gaussian Naïve Bayes, Multinomial Naïve Bayes, Bernoulli Naïve Bayes, and Complement Naïve Bayes) were evaluated to identify the best performing algorithm for each cohort. We simplistically averaged hyperparameter values across the 5 folds to select the final values for the survival analysis; the final hyperparameters chosen for the models in the 3 cohorts are presented in Supplementary Table 6.

## Calibration curve

Calibration curves compares how well the probabilistic predictions of a binary classifier are calibrated.^3, 4^ A one-versus-rest approach was employed to split our multi-class classification dataset into five binary classification problems, with each having a unique hypertension group as the positive class. To produce the calibration curve for each class, the probabilistic predictions were binned into 10 groups of equal size based on the predicted positive class probabilities. For each bin, a point was plotted with the proportion of samples whose class is the positive class on the y-axis and the midpoint of the bin (average predicted probability) on the x-axis. In total, five calibration curves corresponding to five hypertension groups were produced. The Brier score for each group was also calculated by measuring the mean squared difference between the predicted probability and the actual outcome. The lower the Brier score, the better is the skill of the model in detecting the positive class. A Brier score of 0 means perfect accuracy, and a Brier score of 1 means perfect inaccuracy.^4^ The final calibration curves and Brier scores for each ML model were the average of five cross-validation folds. All calculations were carried out using Python and sklearn’s calibration_curve and brier_score_loss functions.

## Evaluation Criteria

We used a confusion matrix of the classification results to compute the performance indices. Based on this confusion matrix, we obtained the following indicators to evaluate the performance of our model.^4, 8^ Accuracy was calculated as the proportion of the correct number of samples (true positives [TP]; the true category of the sample is positive and the final predicted result is also positive) to the total number of samples, including true positives, false negatives (FN; the true category of the sample is positive and the final predicted result is negative), true negatives (TN; the true category of the sample is negative and the final predicted result is also negative), and false positives (FP; the true category of the sample is negative and the final predicted result is positive) using the following formula: TP+TN/TP+FP+TN+FN. Sensitivity, also called recall, was calculated as the percentage of TP examples that were correctly predicted: TP/TP+FN. The positive predictive value (PPV), also known as precision, was calculated as the percentage of positive samples that are predicted correctly: TP/TP+FP. Specificity was calculated as the proportion of TN samples that was correctly predicted: TN/TN+FP. The negative predictive value (NPV) was calculated as the percentage of the sample predicted correctly as a negative example: TN/TN+FN. Finally, the F1-score was calculated as a harmonic average of model accuracy and recall according to the following formula:

2×(precision×recall)/(precision+recall)

We then sorted the samples according to the prediction results of the model, and predicted the samples as positive examples one by one, successively obtaining the FP rate and TP rate, which were plotted as the horizontal and vertical coordinates to obtain the receiver operating characteristic curve (ROC). The area under the ROC curve (AUROC) were subsequently calculated to measure of the ability of the model to distinguish between classes. The AUROC values range from 0 to 1. Since this was a multiclass classification problem, the ROC curve and AUROC scores were performed using a one-versus-rest approach, which involves splitting the multi-class data into multiple binary classification problems. In total, five ROC curves and AUROC scores were generated, corresponding to this research’s five hypertension classes. Finally, a macro average ROC curve and the associated average AUROC score was produced by taking the average of curves across all classes. For each ML model, the ROC curves were averaged across 5 cross-validation folds to give the final curves and AUCs. All calculations and plotting were carried out using Python and sklearn’s roc_curve and roc_auc_score functions

## Feature Importance

For the XGBoost model, feature importance was computed automatically using the built-in attribute of Python’s XGBoost package. Feature importance of the RF and DT models were calculated using scikit-learn’s built-in impurity-based method. Each DT model recursively split the dataset into several smaller sub-datasets based on a feature. The feature importance of SVM and MLR models were inferred by extracting and ranking their coefficients using scikit-learn’s method.

# Data availability

The training data from this study are accessible through the data access procedures of each study center upon request. Access may be granted to those who meet the criteria for confidential access, but data governance regulations prevent the data from being accessible to the public. The leads for each cohort (SP, KN, and ID) can provide guidance on data access procedures, which may necessitate separate applications to the respective health boards. The three XGBoost models, corresponding to three cohorts used in this study have been made freely available at <https://github.com/Tran031194/abpmML>.

# Supplementary Figures

## Supplementary Figure 1:

Receiver operating characteristic (ROC) curves for XGB models in Glasgow cohort (n=923) (A), Gdańsk cohort (n=709) (B) and Birmingham cohort (n=1,222) (C). AUROC: area under the curve; kNN: K-nearest neighbours; SVM: support vector machine. All AUROC presented with ±SD.


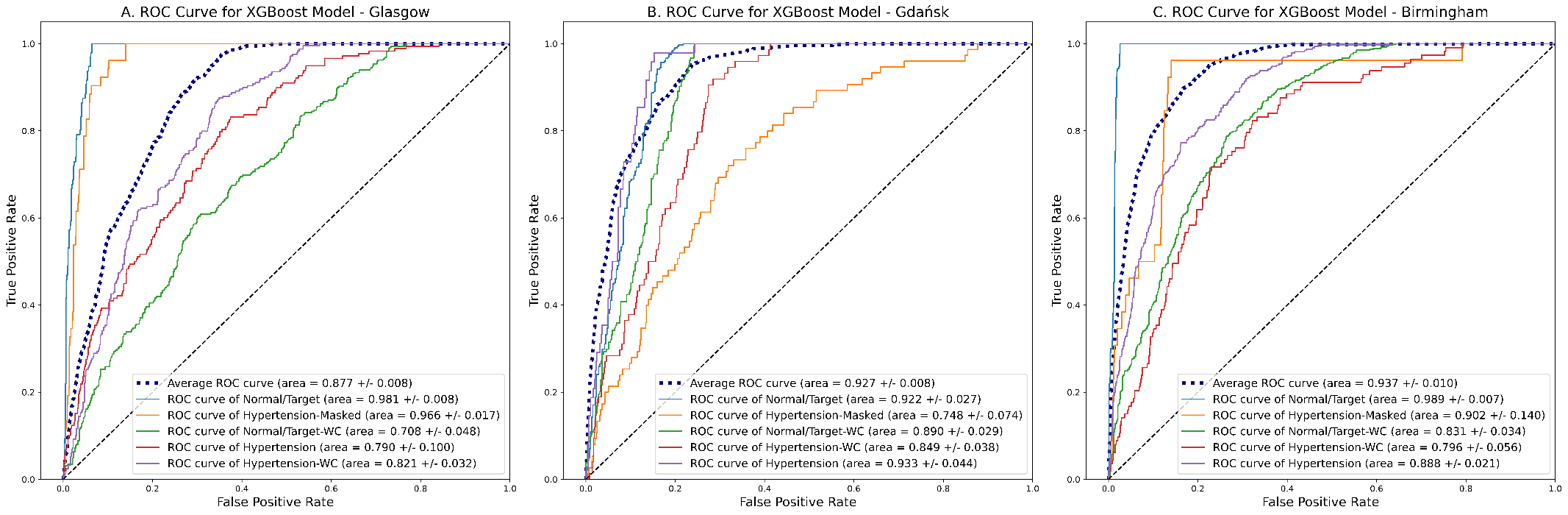


## Supplementary Figure 2:

Receiver operating characteristic (ROC) curves for 7 classification models for a) the Glasgow cohort, b) the Gdańsk cohort and c) the Birmingham cohort. MLR: multinomial logistic regression; SVM: support vector machine; kNN: k-nearest neighbor; NB: naïve Bayes; DT: decision tree; RF: random forest; XGB: tree-based extreme gradient boosting; ROC: receiver operating characteristic; AUROC: area under the receiver operating characteristic curve. All AUROCs presented with ±SD.

(a)


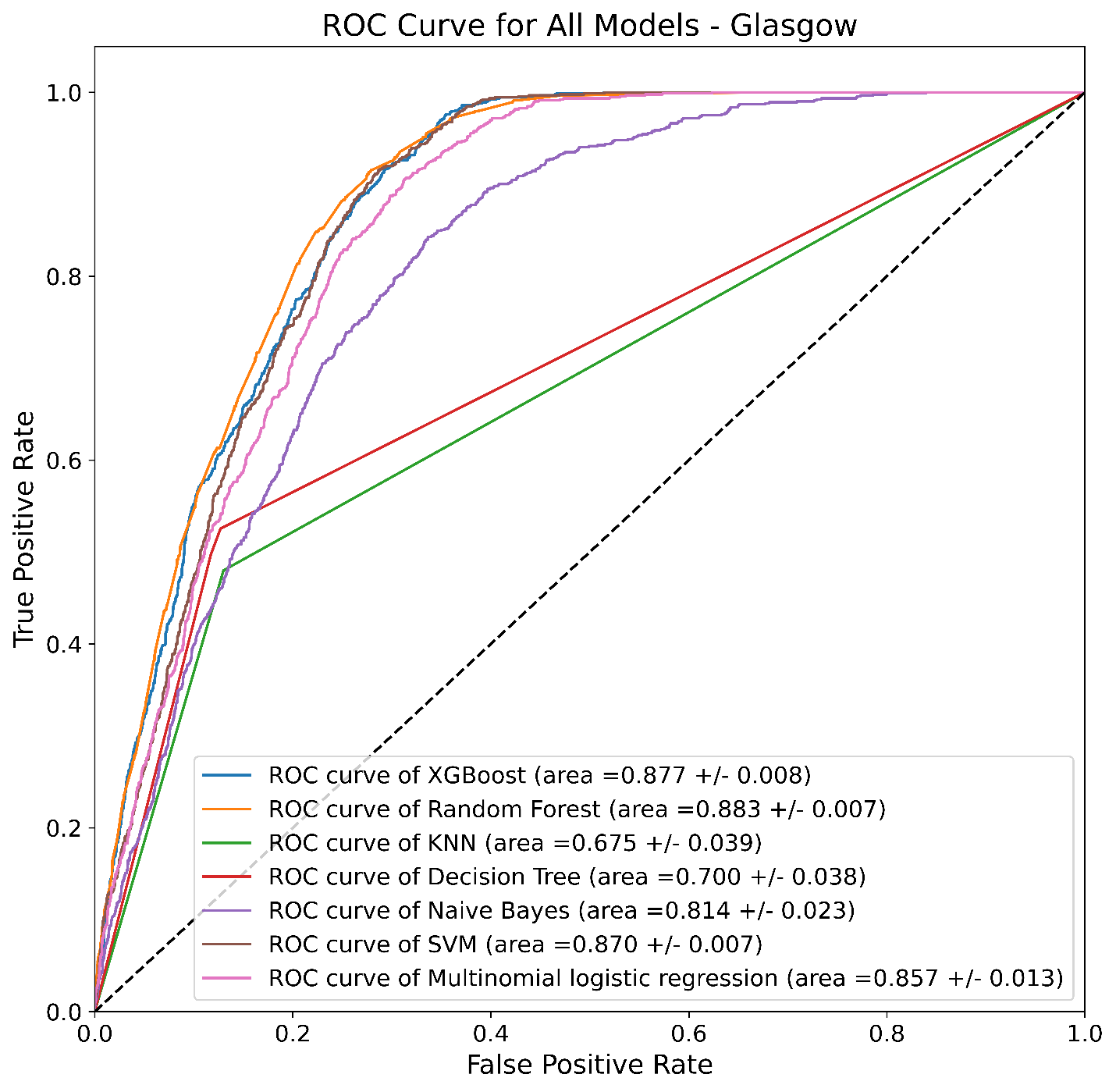


(b)


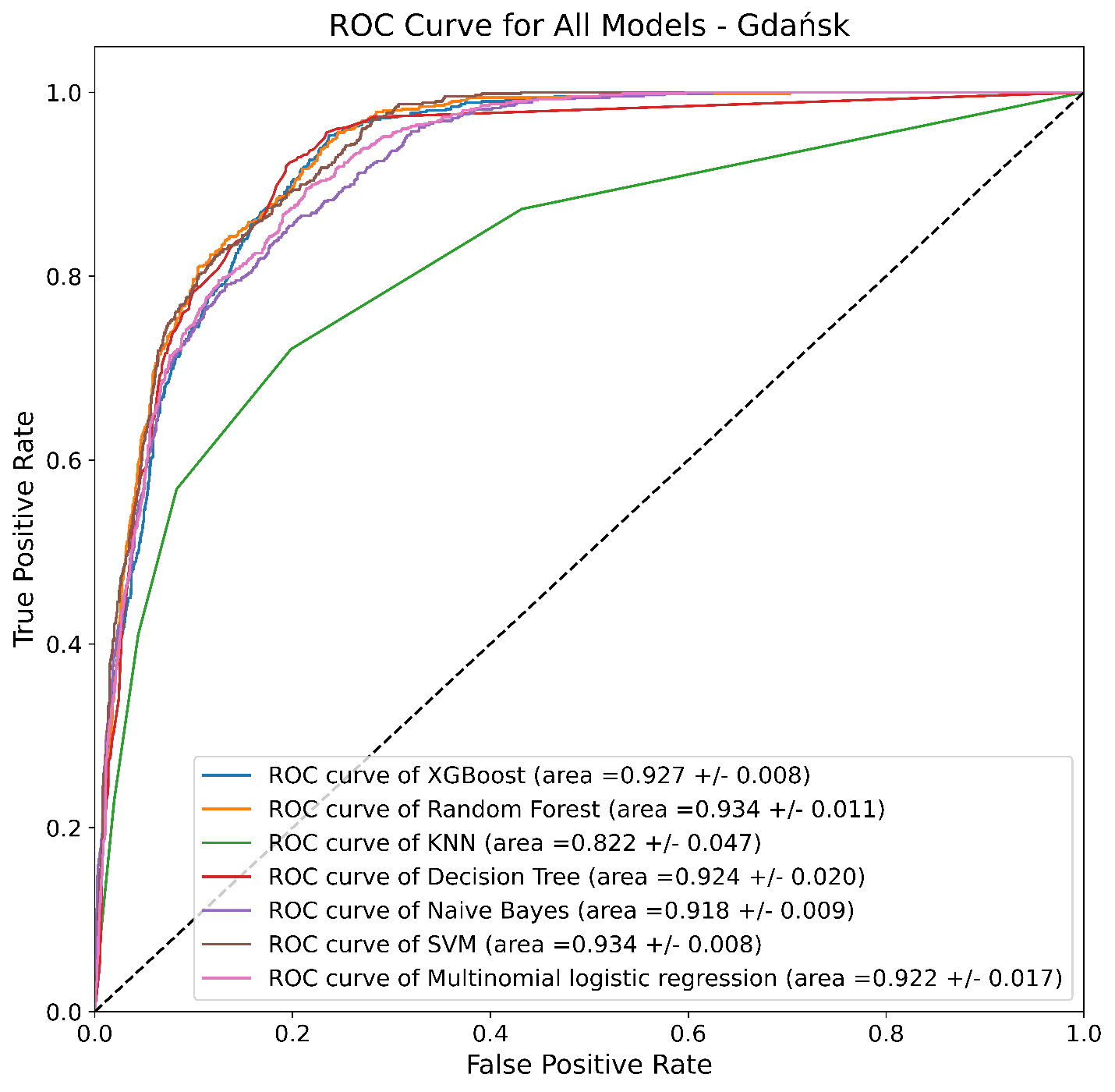


(a)

(c)


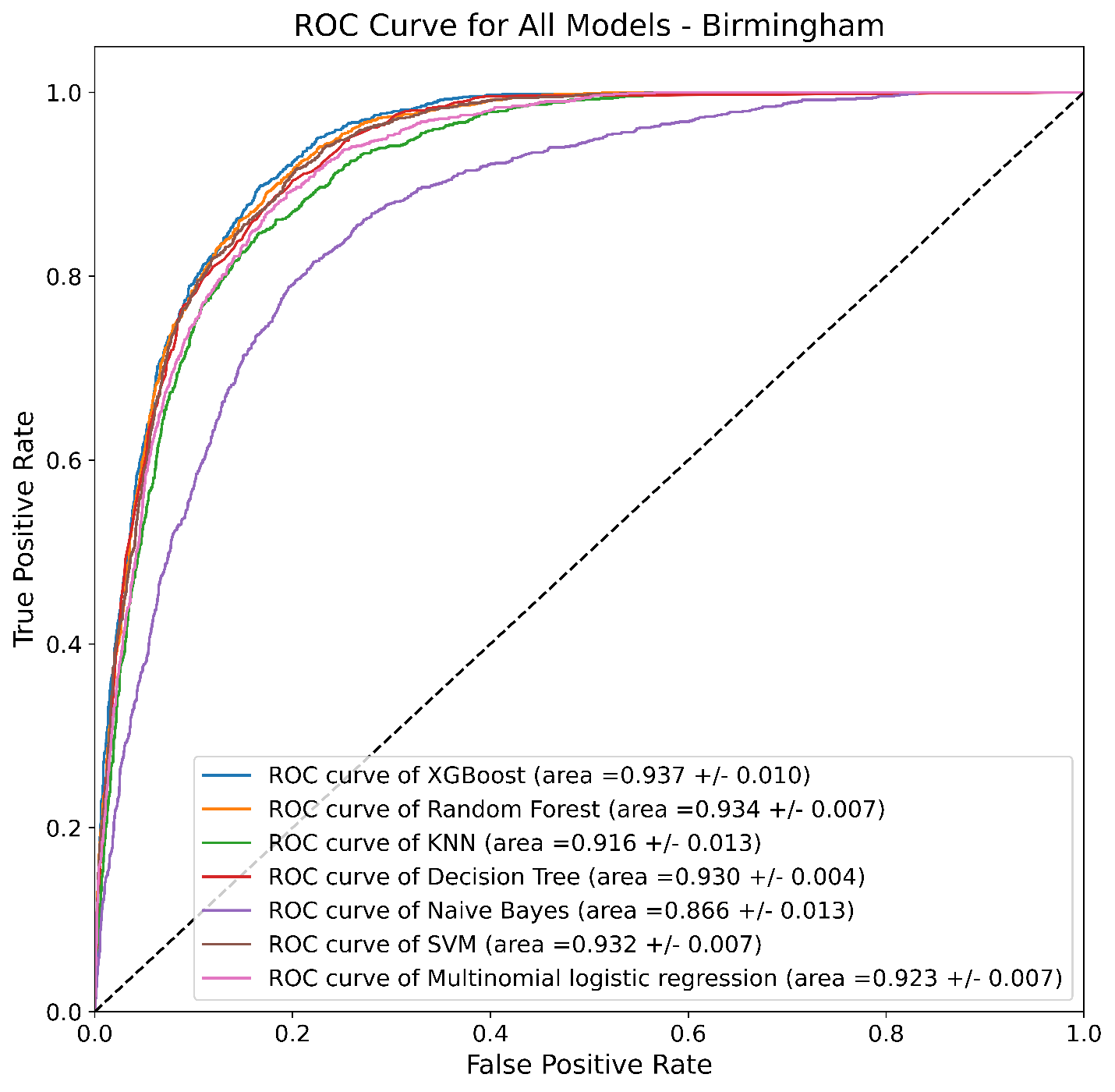


## Supplementary Figure 3:

Confusion matrices for machine learning models developed on the Glasgow dataset (n=923). A: XGB, B: kNN, C: DT, D: RF, E: NB, F: SVM, G: MLR models. WC: white-coat.


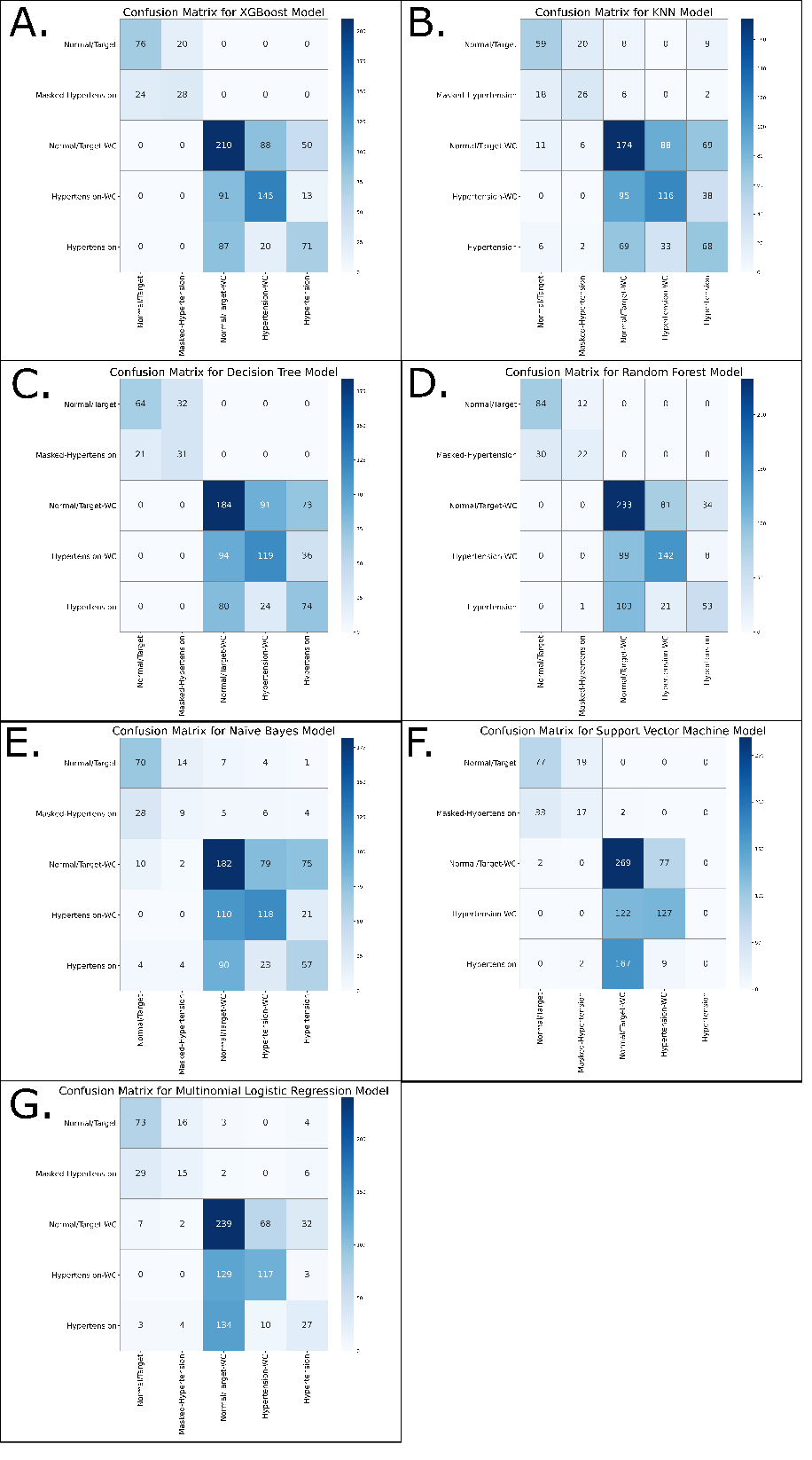


## Supplementary Figure 4:

Calibration plots for the XGBoost model for a) the Glasgow cohort, b) the Gdańsk cohort and c) the Birmingham cohort. Brier scores presented with ±SD. WC: white-coat.

(a)


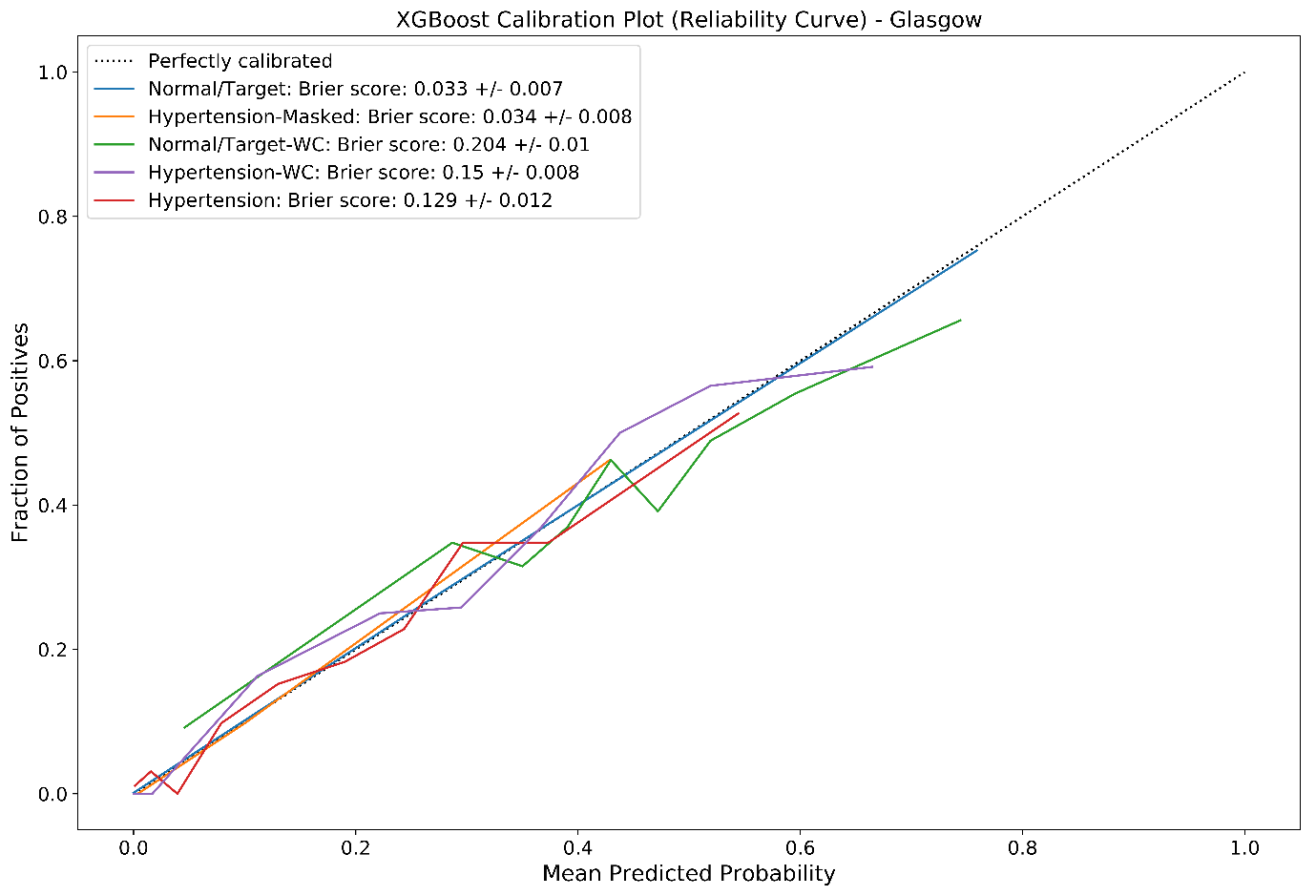


(b)


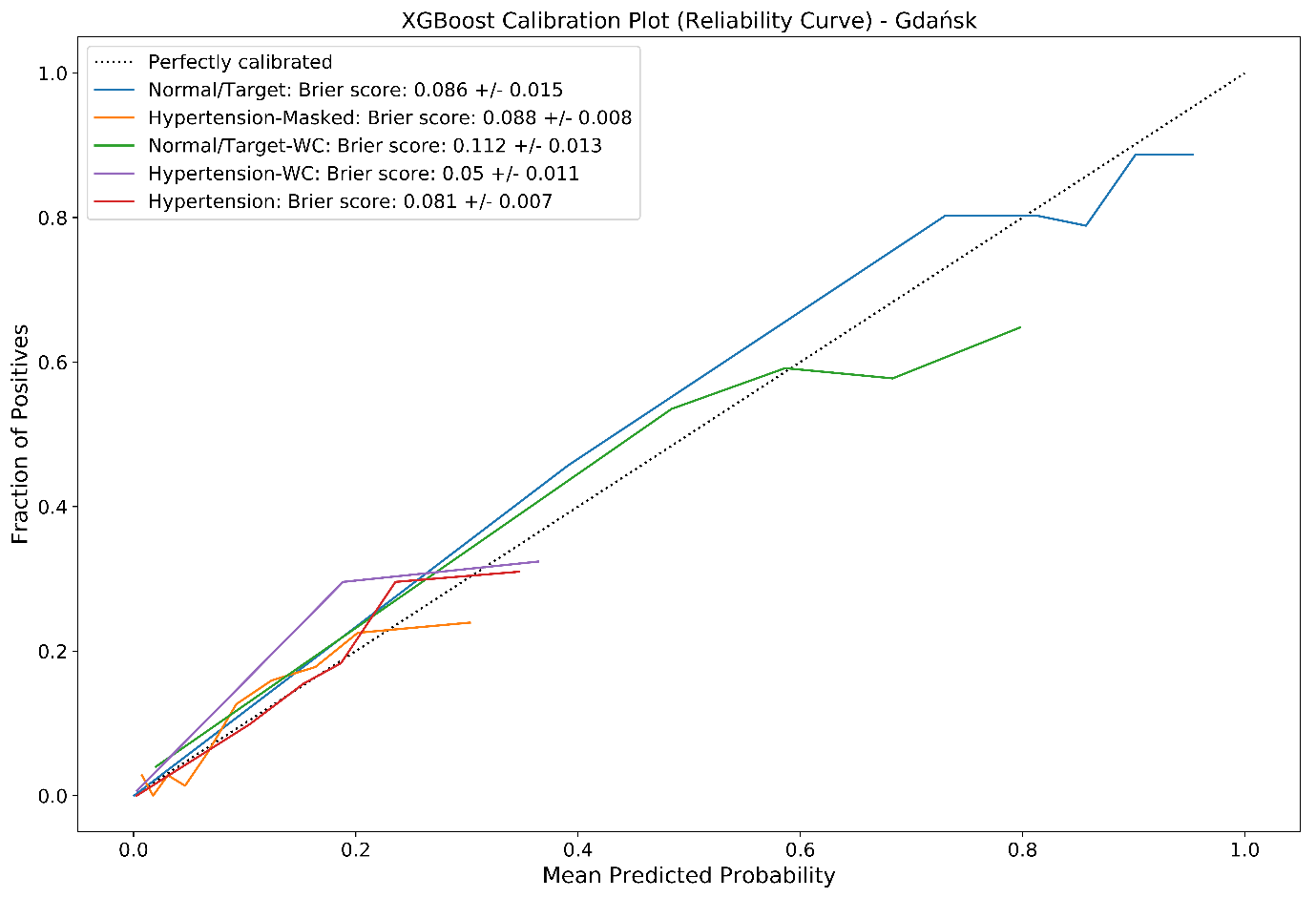


(c)


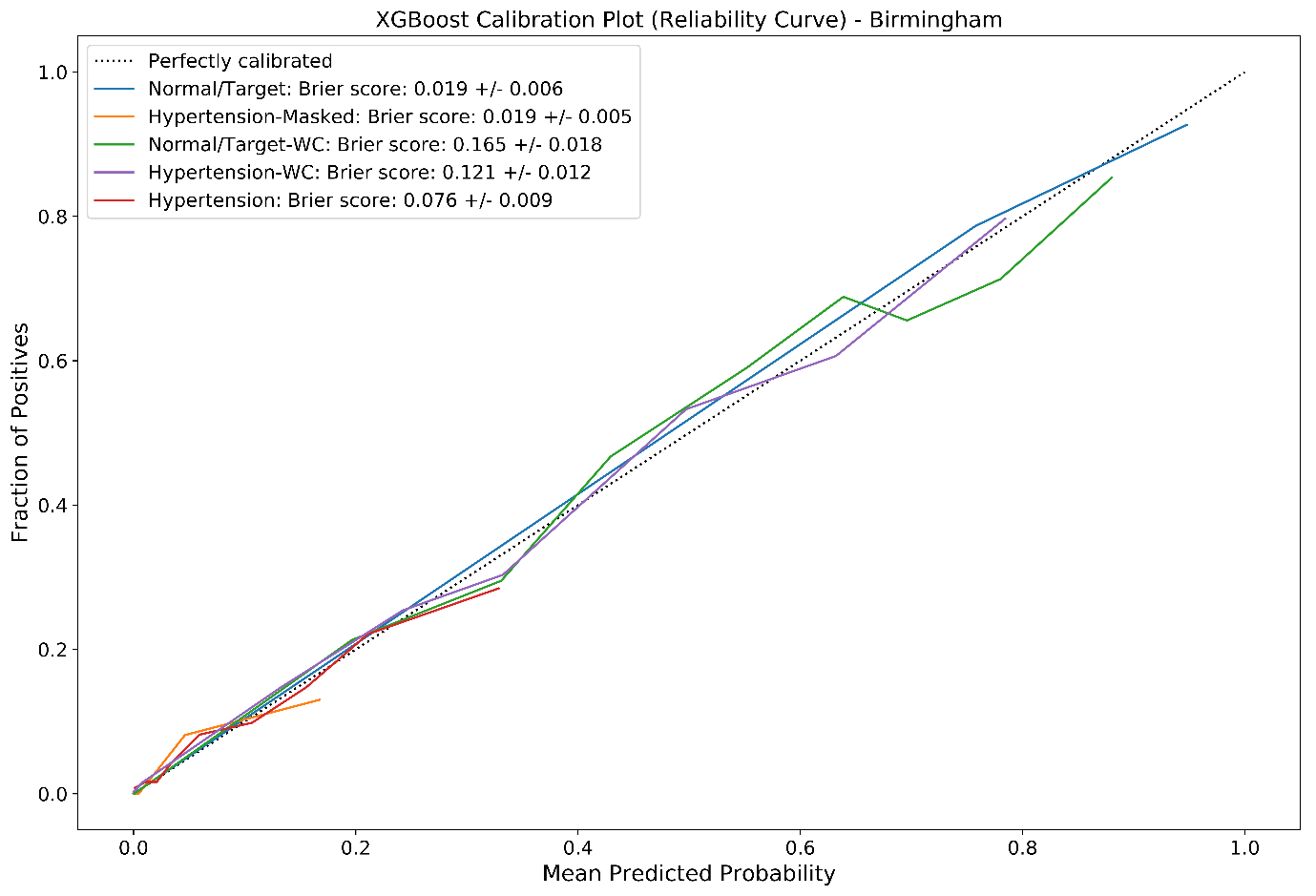


## Supplementary Figure 5:

XGBoost model feature importance ranking for Glasgow (A), Gdańsk (B) and Birmingham (C) cohorts with F importance scores. ALT: alanine aminotransferase; CVD: cardiovascular disease; DBP: diastolic blood pressure; HTN: hypertension; oBP: office blood pressure; SBP: systolic blood pressure.


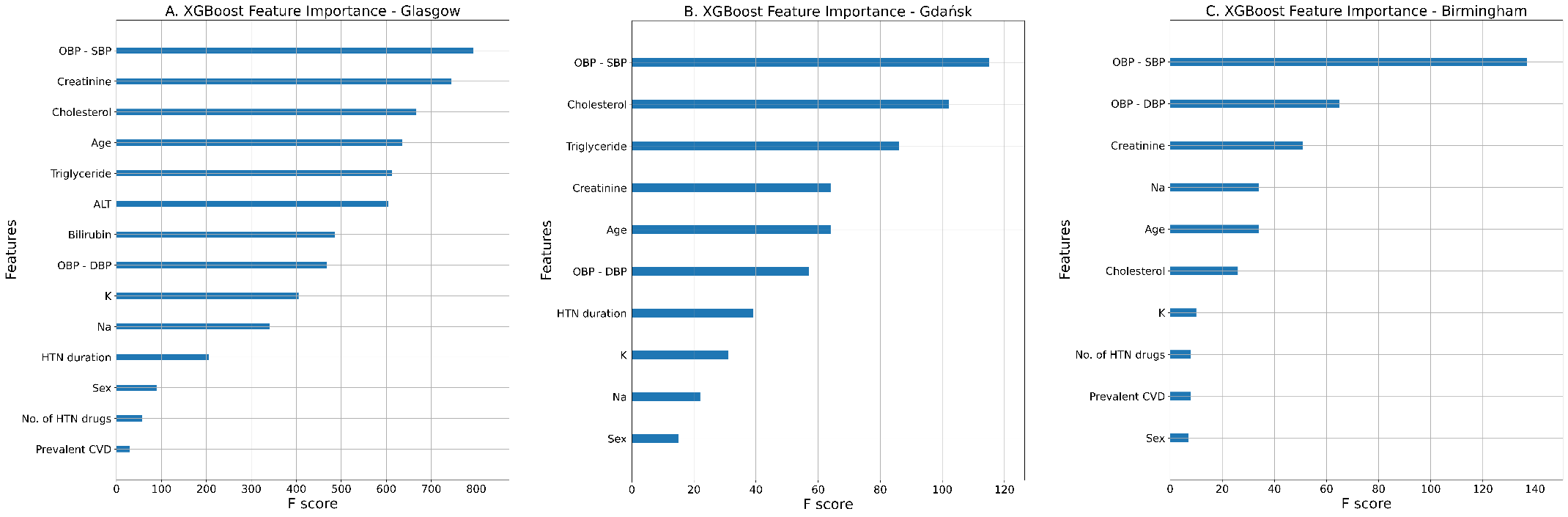


## Supplementary Figure 6:

Feature importance for the 7 classification models for a) the Glasgow cohort, b) the Gdańsk cohort and c) the Birmingham cohort. ALT: alanine aminotransferase; CVD: cardiovascular disease; DBP: diastolic blood pressure; HTN: hypertension; oBP: office blood pressure; SBP: systolic blood pressure; SVM: support vector machine.


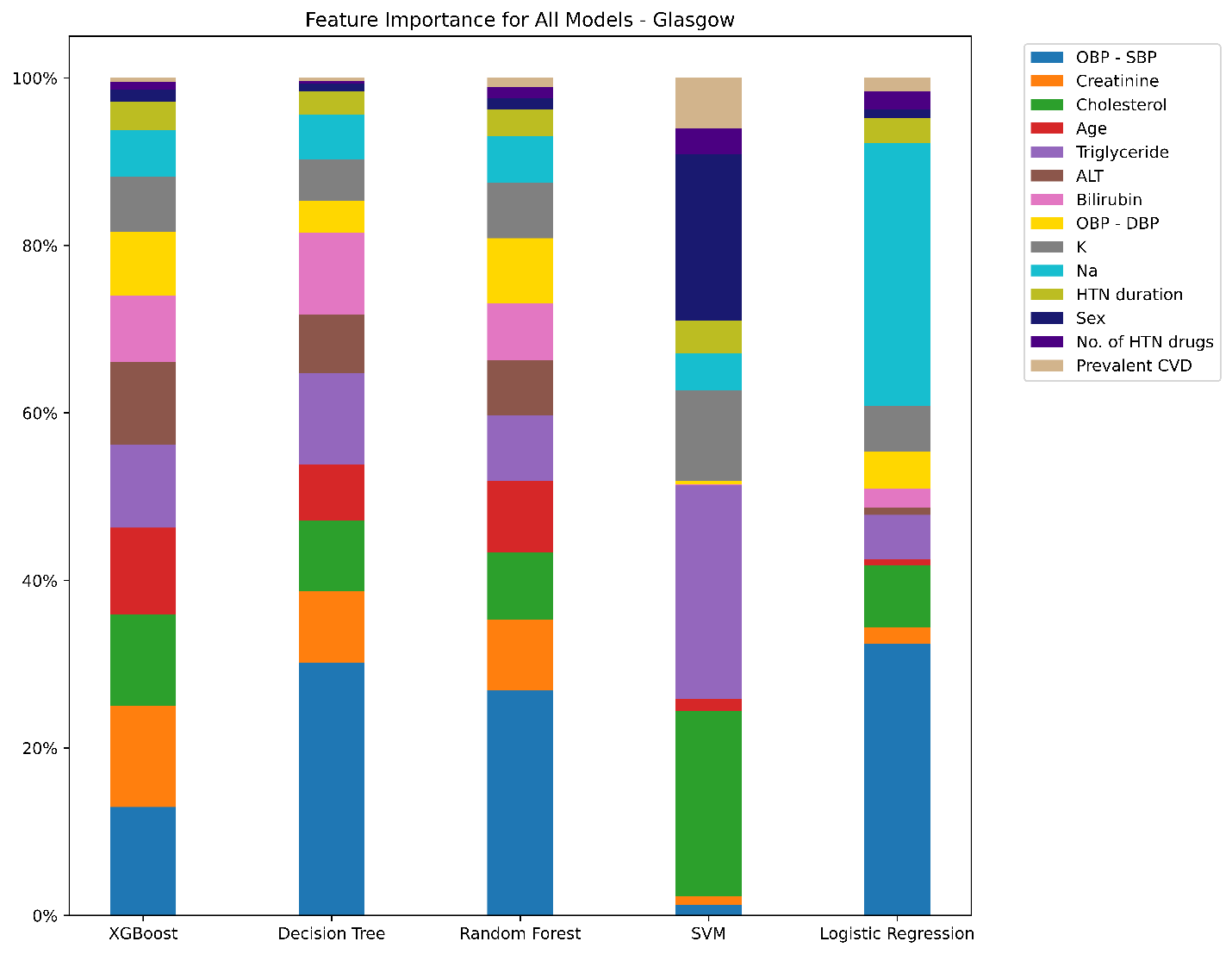


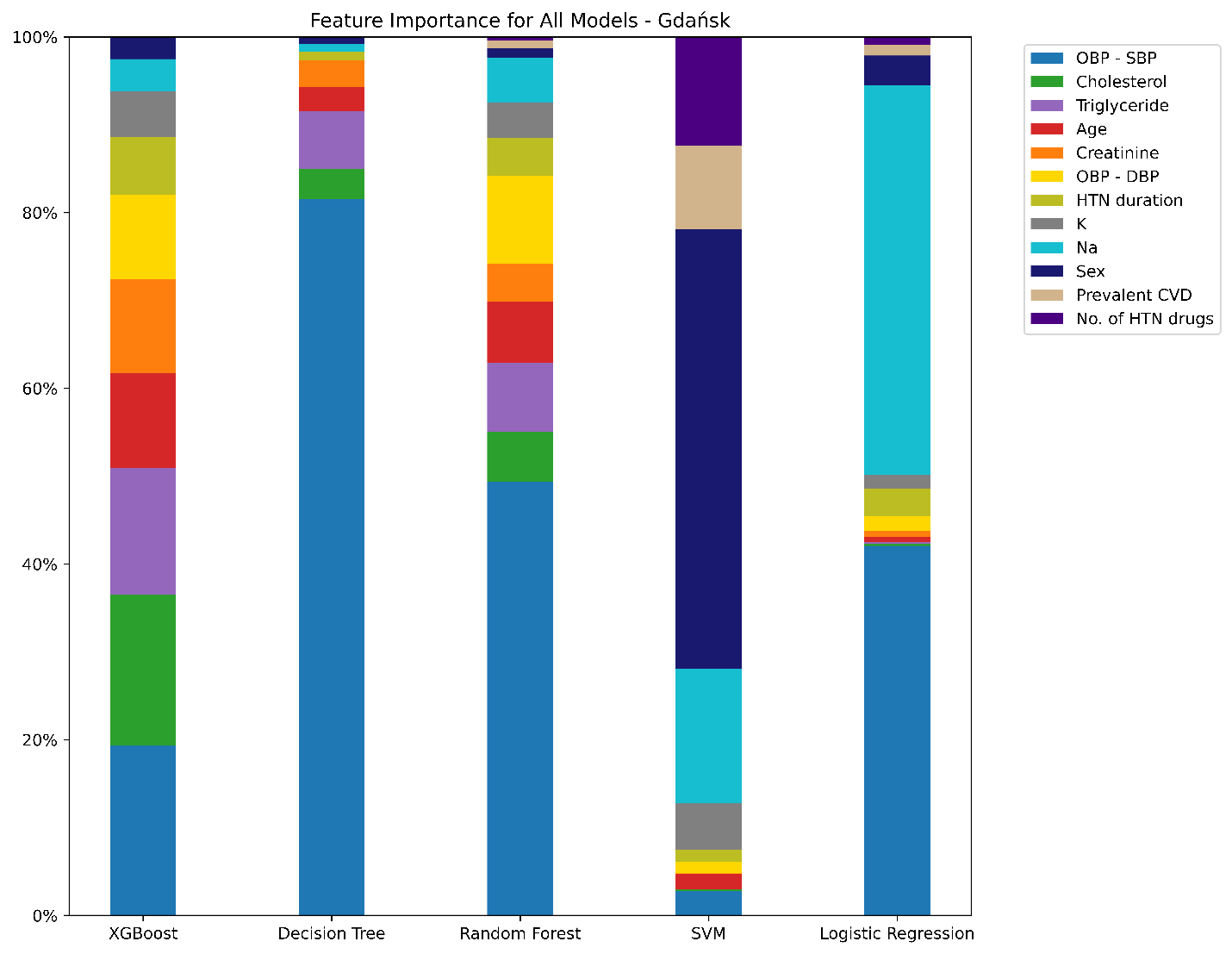


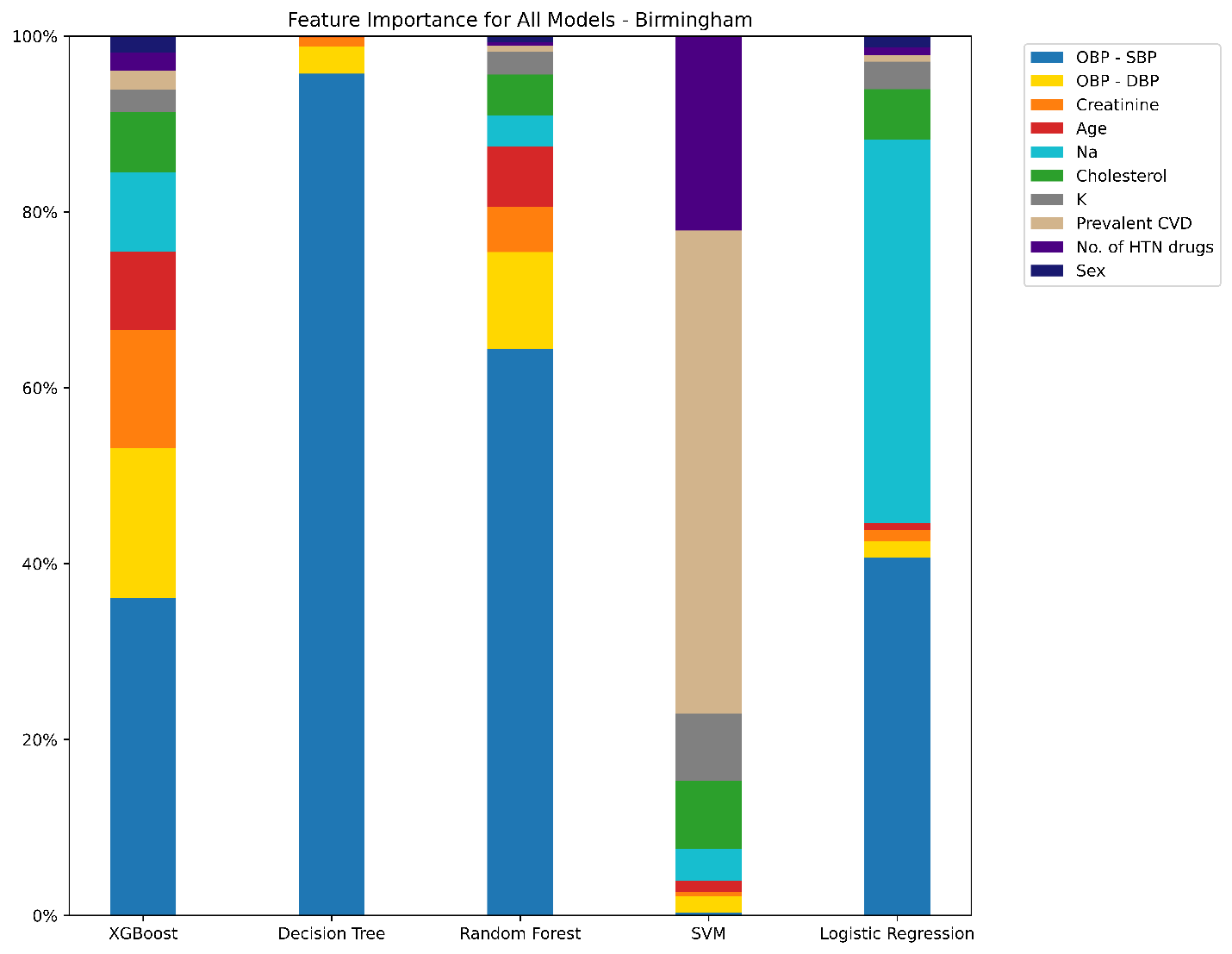


## Supplementary Figure 7:

**
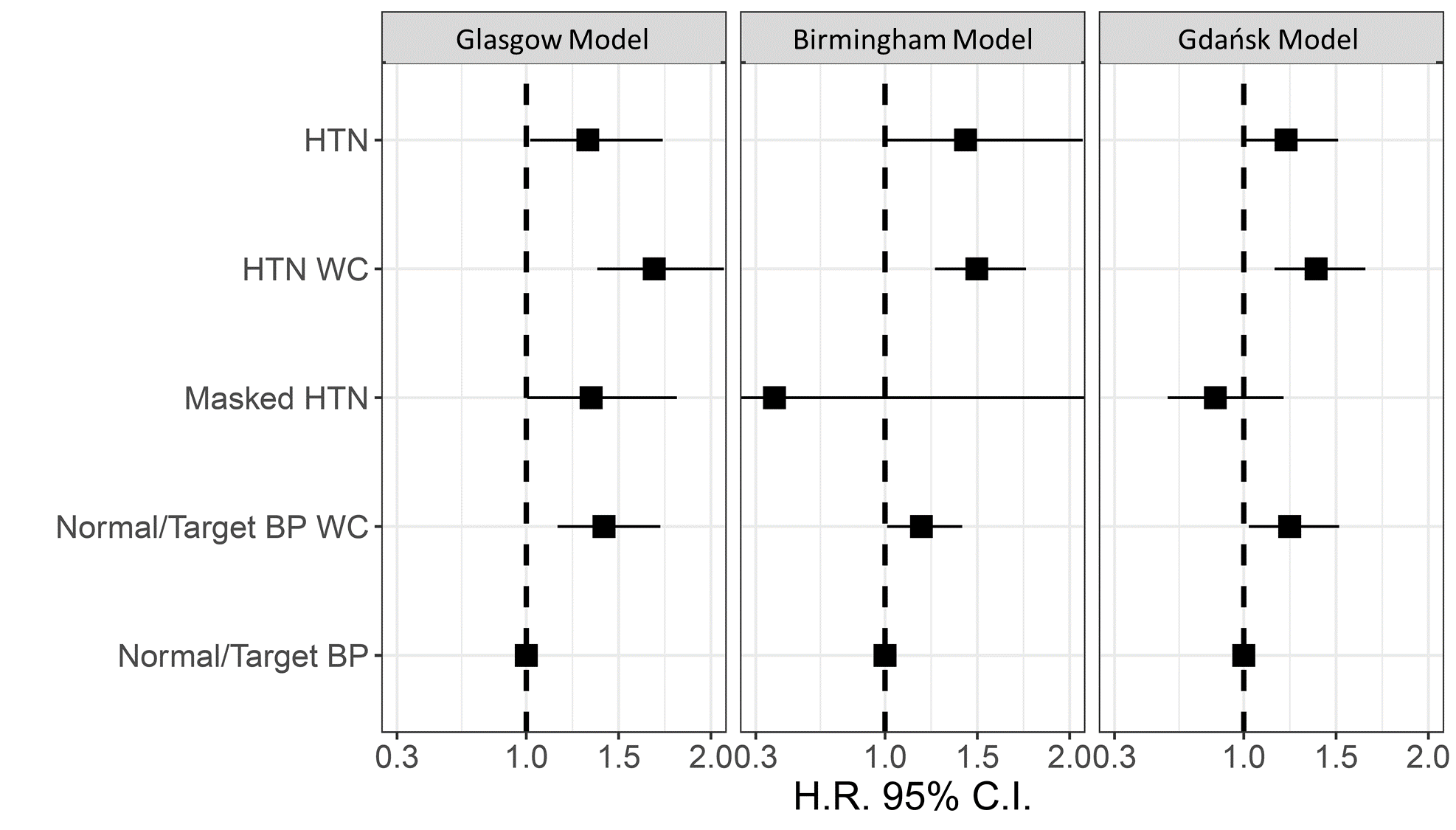
**Forest plot of hazard ratios (HRs) with 95% CI for a) 27-year all-cause mortality and b) 10-year composite cardiovascular events by BP groups inferred using XGB models trained on Glasgow, Birmingham and Gdańsk cohorts.

**(a)**

**
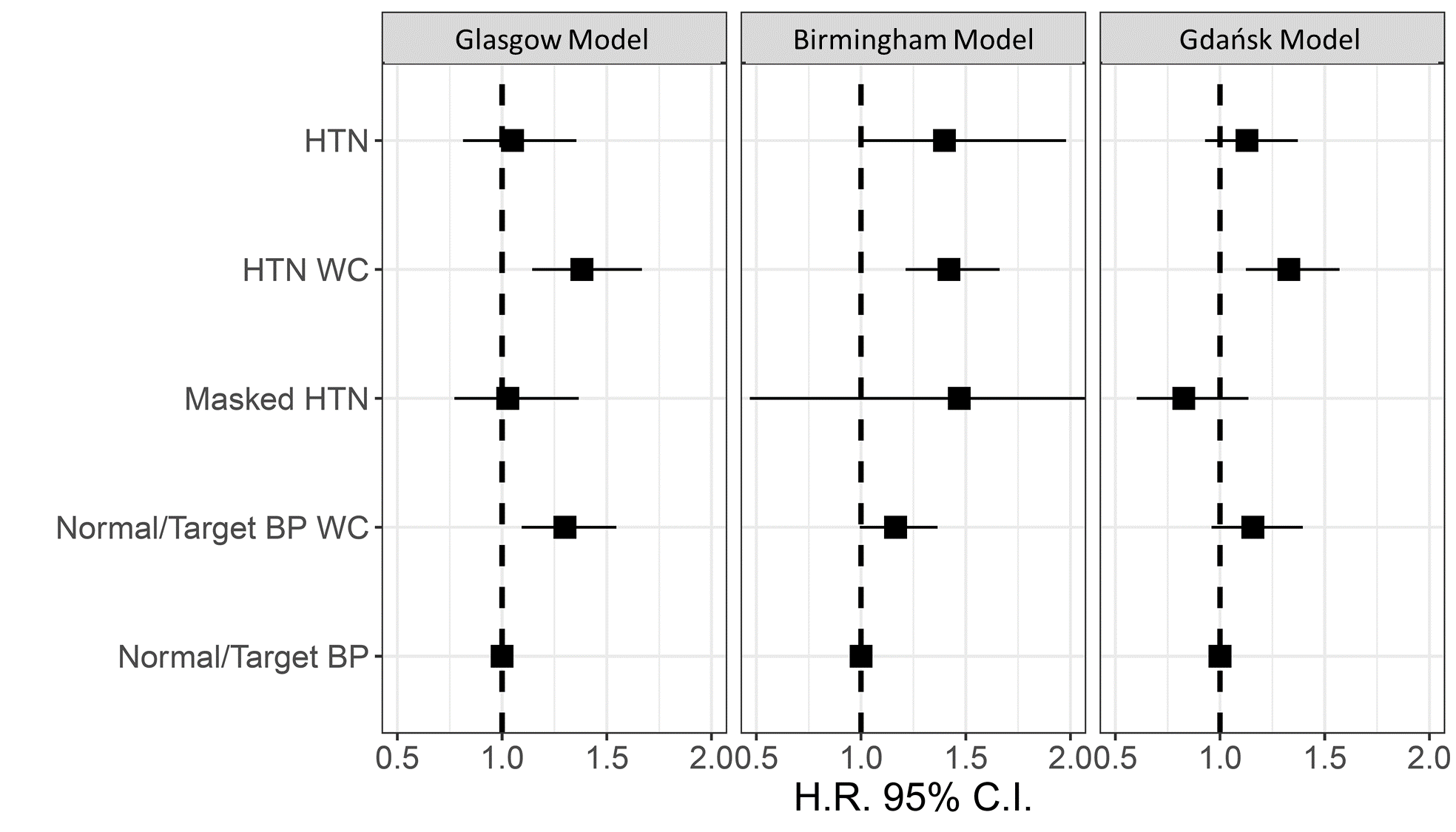
(b)**

# Supplementary Tables

## Supplementary Table 1:

Clinical features used to train machine learning models for the three patient cohorts. AUROC: area under the receiver operating characteristic curve.

| **Feature Name** | **Glasgow** | **Gdańsk** | **Birmingham** |
| --- | --- | --- | --- |
|  | **n=923** | **n=709** | **n=1,222** |
| **Age (years)** | X | X | X |
| **Sex (% female)** | X | X | X |
| **oBP - SBP (mmHg)** | X | X | X |
| **oBP - DBP (mmHg)** | X | X | X |
| **ABPM - SBP (mmHg)** | X | X | X |
| **ABPM - DBP (mmHg)** | X | X | X |
| **Hypertension Duration (years)** | X | X | - |
| **Prevalent CVD (%)** | X | X | X |
| **Antihypertensive Treatment (% treated)** | X | X | X |
| **ALT (IU/L)** | X | - | - |
| **Bilirubin (μmol/L)** | X | - | - |
| **Cholesterol (mmol/L)** | X | X | X |
| **Creatinine (μmol/L)** | X | X | X |
| **Potassium (mmol/L)** | X | X | X |
| **Sodium (mmol/L)** | X | X | X |
| **Triglyceride (mmol/L)** | X | X | - |

**Table 1:** Definition of five BP groups. aSBP: ABPM 24-hr systolic blood pressure average; BP: blood pressure; oSBP: office systolic blood pressure; WC: white-coat.

| **BP Group** | **oSBP (mmHg)** | **aSBP (mmHg)** | **oSBP-aSBP (mmHg)** |
| --- | --- | --- | --- |
| Normal/Target | ≤140 | ≤135 | ·· |
| Hypertension-Masked | ≤140 | >135 | ·· |
| Normal/Target-WC | >140 | ≤135 | ·· |
| Hypertension-WC | >140 | >135 | ≥15 |
| Hypertension | >140 | >135 | ·· |

**Table 2:** Summary of patient characteristics presented as mean ±SD, unless presented as proportion (%) of total cohort. Distribution of patients across five BP groups is also shown. Grey shading indicates absence of data. ABPM: ambulatory blood pressure monitoring; ALT: alanine aminotransferase; CVD: cardiovascular disease; DBP: systolic blood pressure; oBP: office blood pressure; SBP: systolic blood pressure; WC: white-coat.

|  | **Glasgow** | **Gdańsk** | **Birmingham** |
| --- | --- | --- | --- |
|  | **n=923** | **n=709** | **n=1,222** |
| **Demographics** | | | |
| **Age (years)** | 50·7±16·3 | 54·4±13·1 | 55·7±13·9 |
| **Sex (% female)** | 43·1 | 45·6 | 56·2 |
| **BP Measurements & Hypertension Status** | | | |
| **oBP – SBP (mmHg)** | 163·1±22·5 | 139·9±19·5 | 157·4±19·9 |
| **oBP – DBP (mmHg)** | 95·4±14·3 | 82·2±11·2 | 94·6±12·0 |
| **ABPM - SBP (mmHg)** | 138·0±15·9 | 129·1±12·3 | 132·3±13·1 |
| **ABPM - DBP (mmHg)** | 81·5±11·5 | 77·3±8·9 | 78·5±9·7 |
| **Hypertension Duration (years)** | 3·7±4·7 | 12·1±8·9 | ·· |
| **Prevalent CVD (%)** | 56·6 | 16·2 | 7·8 |
| **Antihypertensive Treatment (% treated)** | 54·0 | 96·1 | 42·2 |
| **BP Groups** | | | |
| **Normal/Target** | 96 (10·4%) | 328 (46·3%) | 210 (17·2%) |
| **Hypertension-Masked** | 52 (5·6%) | 75 (10·6%) | 26 (2·1%) |
| **Normal/Target-WC** | 348 (37·7%) | 184 (26·0%) | 547 (44·8%) |
| **Hypertension-WC** | 249 (27·0%) | 48(6·8%) | 326 (26·7%) |
| **Hypertension** | 178 (19·3%) | 74 (10·4%) | 113 (9·2%) |
| **Blood Chemistry** | | | |
| **Sodium (mmol/L)** | 139·4+2·5 | 140·0±2·5 | 140·1±2·7 |
| **Potassium (mmol/L)** | 4·4±0·4 | 4·2±0·4 | 4·3±0·4 |
| **Creatinine (μmol/L)** | 75·7±19·3 | 73·6±13·1 | 80·7±17·7 |
| **Cholesterol (mmol/L)** | 5·4±1·2 | 5·0±1·1 | 5·4±1·1 |
| **Triglyceride (mmol/L)** | 1·9±1·2 | 1·5±0·8 | ·· |
| **ALT (U/L)** | 28·5±18·6 | ·· | ·· |
| **Bilirubin (μmol/L)** | 10·5±5·3 | ·· | ·· |

## Supplementary Table 2:

Patient characteristics by hypertension class for Glasgow (n=923) cohort at hypertension threshold = 135 mmHg. ABPM: ambulatory blood pressure monitoring; ALT: alanine aminotransferase; CVD: cardiovascular disease; DBP: systolic blood pressure; oBP: office blood pressure; SBP: systolic blood pressure; WC: white-coat.

| **Glasgow (n=923)** | **Normal/Target** | **Hypertension- Masked** | **Normal/Target-WC** | **Hypertension-WC** | **Hypertension** |
| --- | --- | --- | --- | --- | --- |
|  | **n=96** | **n=52** | **n=348** | **n=249** | **n=178** |
| **Age (years)** | 43.7±14.9 | 51.7±14.5 | 49.6±16.1 | 55.7±15.8 | 49.4±16.5 |
| **oBP - SBP (mmHg)** | 131.9±8.1 | 133.9±6.8 | 165.6±17.3 | 181.6±20.2 | 157.7±11.7 |
| **oBP - DBP (mmHg)** | 87.7±12.2 | 86.2±11.3 | 96.7±13.8 | 98.6±16.0 | 95.0±12.2 |
| **ABPM - SBP (mmHg)** | 124.0±7.8 | 145.5±8.5 | 125.9±7.4 | 147.3±11.1 | 154.0±14.1 |
| **ABPM - DBP (mmHg)** | 77.7±8.0 | 86.7±10.6 | 75.6±8.5 | 83.8±11.0 | 90.5±11.7 |
| **Hypertension Duration (years)** | 2.1±3.8 | 4.6±5.0 | 3.4±4.5 | 4.5±4.9 | 4.0±4.8 |
| **ALT (IU/L)** | 26.8±16.9 | 34.3±20.8 | 28.3±17.9 | 28.7±20.3 | 27.7±18.0 |
| **Bilirubin (μmol/L)** | 10.0±5.6 | 11.1±4.9 | 10.6±4.9 | 10.1±5.3 | 11.0±5.8 |
| **Cholesterol (mmol/L)** | 5.3±1.3 | 5.4±1.4 | 5.5±1.2 | 5.3±1.2 | 5.4±1.3 |
| **Creatinine (μmol/L)** | 72.5±21.2 | 77.3±21.4 | 76.6±19.6 | 74.1±15.1 | 77.6±21.7 |
| **Potassium (mmol/L)** | 4.5±0.5 | 4.4±0.4 | 4.4±0.4 | 4.4±0.4 | 4.4±0.5 |
| **Sodium (mmol/L)** | 139.2±2.5 | 139.4±2.9 | 139.4±2.4 | 139.4±2.5 | 139.4±2.3 |
| **Triglyceride (mmol/L)** | 1.9±1.2 | 2.2±1.3 | 2.0±1.4 | 1.7±1.0 | 1.8±1.2 |

## Supplementary Table 3:

Patient characteristics by hypertension class for Gdańsk (n=709) cohort at hypertension threshold = 135 mmHg. ABPM: ambulatory blood pressure monitoring; ALT: alanine aminotransferase; CVD: cardiovascular disease; DBP: systolic blood pressure; oBP: office blood pressure; SBP: systolic blood pressure; WC: white-coat.

| **Gdańsk (n=709)** | **Normal/Target** | **Hypertension- Masked** | **Normal/Target-WC** | **Hypertension-WC** | **Hypertension** |
| --- | --- | --- | --- | --- | --- |
|  | **n=328** | **n=75** | **n=184** | **n=48** | **n=74** |
| **Age (years)** | 52.5±13.2 | 51.1±14.2 | 57.0±11.8 | 60.8±10.4 | 55.8±13.3 |
| **oBP - SBP (mmHg)** | 126.2±9.3 | 130.3±8.9 | 154.0±13.5 | 177.4±17.8 | 151.1±8.7 |
| **oBP - DBP (mmHg)** | 77.4±8.5 | 78.7±9.4 | 87.4±9.9 | 91.8±14.6 | 87.4±12.6 |
| **ABPM - SBP (mmHg)** | 122.0±7.5 | 140.7±5.0 | 125.2±6.9 | 145.6±8.9 | 147.2±10.4 |
| **ABPM - DBP (mmHg)** | 74.8±7.3 | 84.3±7.8 | 74.6±7.5 | 80.7±8.4 | 85.8±10.2 |
| **Hypertension Duration (years)** | 11.5±8.9 | 12.2±9.1 | 12.5±8.2 | 14.3±9.0 | 12.4±9.7 |
| **Cholesterol (mg/dL)** | 191.9±40.1 | 191.6±39.1 | 198.7±40.4 | 196.4±49.5 | 200.3±41.7 |
| **Creatinine (μmol/L)** | 72.8±13.4 | 75.1±12.7 | 73.6±12.9 | 73.7±13.6 | 74.8±12.9 |
| **Potassium (mmol/L)** | 4.3±0.4 | 4.2±0.4 | 4.3±0.4 | 4.3±0.4 | 4.2±0.4 |
| **Sodium (mmol/L)** | 139.9±2.5 | 140.3±2.4 | 140.0±2.4 | 140.2±2.6 | 139.7±2.8 |
| **Triglyceride (mg/dL)** | 124.7±62.2 | 137.3±78.3 | 137.3±63.0 | 129.1±61.3 | 152.5±84.3 |

## Supplementary Table 4:

Patient characteristics by hypertension class for Birmingham (n=1,222) cohort at hypertension threshold = 135 mmHg. ABPM: ambulatory blood pressure monitoring; ALT: alanine aminotransferase; BP: blood pressure; CVD: cardiovascular disease; DBP: systolic blood pressure; oBP: office blood pressure; SBP: systolic blood pressure; WC: white-coat.

| **Birmingham (n=1,222)** | **Normal/Target** | **Hypertension- Masked** | **Normal/Target-WC** | **Hypertension-WC** | **Hypertension** |
| --- | --- | --- | --- | --- | --- |
|  | **n=210** | **n=26** | **n=547** | **n=326** | **n=113** |
| **Age (years)** | 48.0±14.4 | 51.7±12.4 | 56.7±13.1 | 59.4±13.2 | 54.9±14.1 |
| **oBP - SBP (mmHg)** | 130.6±8.8 | 132.0±11.9 | 158.0±13.0 | 176.5±15.1 | 154.4±9.9 |
| **oBP - DBP (mmHg)** | 86.6±10.0 | 90.7±19.4 | 94.2±10.3 | 99.8±11.8 | 96.7±13.5 |
| **ABPM - SBP (mmHg)** | 120.4±8.0 | 141.7±7.6 | 125.7±6.6 | 144.6±8.8 | 148.5±9.8 |
| **ABPM - DBP (mmHg)** | 75.1±7.2 | 86.5±11.6 | 75.2±8.1 | 82.7±9.4 | 87.0±10.4 |
| **Cholesterol (mmol/L)** | 5.3±1.2 | 5.2±1.3 | 5.5±1.1 | 5.4±1.0 | 5.3±1.1 |
| **Creatinine (μmol/L)** | 79.3±18.6 | 78.9±23.2 | 79.6±16.4 | 82.6±17.8 | 83.7±20.4 |
| **Potassium (mmol/L)** | 4.3±0.3 | 4.4±0.3 | 4.3±0.4 | 4.3±0.4 | 4.3±0.4 |
| **Sodium (mmol/L)** | 140.1±2.3 | 140.1±2.9 | 140.2±2.5 | 140.0±3.1 | 140.1±2.5 |

## Supplementary Table 5:

Summary of model performance for the three cohorts. AUROC: area under the receiver operating characteristic curve; k-NN: k-nearest neighbours; MLR: multinomial logistic regression; SVM: support vector machine.

| Cohort | Metric | XGBoost | k-NN | Decision Tree | Random Forest | Naive Bayes | SVM | MLR |
| --- | --- | --- | --- | --- | --- | --- | --- | --- |
| Glasgow (n=923) | Accuracy | 0.574 ± 0.032 | 0.480 ± 0.063 | 0.511 ± 0.053 | 0.579 ± 0.033 | 0.472 ± 0.058 | 0.531 ± 0.038 | 0.511 ± 0.029 |
|  | F1 score | 0.570 ± 0.032 | 0.479 ± 0.063 | 0.511 ± 0.053 | 0.565 ± 0.045 | 0.465 ± 0.060 | 0.470 ± 0.038 | 0.488 ± 0.031 |
|  | AUROC | 0.877 ± 0.008 | 0.675 ± 0.039 | 0.700± 0.038 | 0.883 ± 0.007 | 0.814 ± 0.023 | 0.870 ± 0.005 | 0.857 ± 0.013 |
| Gdańsk (n=709) | Accuracy | 0.729 ± 0.031 | 0.612 ± 0.042 | 0.719 ± 0.045 | 0.735 ± 0.024 | 0.702 ± 0.027 | 0.728 ± 0.024 | 0.702 ± 0.025 |
|  | F1 score | 0.664 ± 0.036 | 0.546 ± 0.041 | 0.678 ± 0.047 | 0.658 ± 0.044 | 0.607 ± 0.032 | 0.638 ± 0.033 | 0.617 ± 0.031 |
|  | AUROC | 0.938 ± 0.004 | 0.822 ± 0.047 | 0.924 ± 0.020 | 0.934 ± 0.011 | 0.918 ± 0.009 | 0.933 ± 0.010 | 0.922 ± 0.017 |
| Birmingham (n=1,222) | Accuracy | 0.724 ± 0.045 | 0.686 ± 0.065 | 0.703 ± 0.045 | 0.721 ± 0.035 | 0.601 ± 0.071 | 0.713 ± 0.026 | 0.685 ± 0.050 |
|  | F1 score | 0.692 ± 0.047 | 0.645 ± 0.067 | 0.661 ± 0.045 | 0.678 ± 0.031 | 0.567 ± 0.080 | 0.666 ± 0.027 | 0.641 ± 0.050 |
|  | AUROC | 0.937 ± 0.010 | 0.916 ± 0.013 | 0.930 ± 0.004 | 0.934 ± 0.007 | 0.866 ± 0.013 | 0.931 ± 0.006 | 0.923 ± 0.007 |

## Supplementary Table 6:

Demographics of Glasgow non-ABPM cohort with predicted BP groups using XGBoost models trained on Glasgow, Birmingham and Gdańsk cohorts respectively. BMI: body mass index; BP: blood pressure; CV: cardiovascular; DBP: diastolic blood pressure; SBP: systolic blood pressure; WC: white-coat.

|  | | | **BP Classes: Glasgow Model** | | | | | |
| --- | --- | --- | --- | --- | --- | --- | --- | --- |
|  | | **Overall** | **Normal/Target** | **Normal/Target-WC** | **Hypertension-Masked** | **Hypertension -WC** | **Hypertension** | **Missing N(%)** |
| **Total N (%)** |  | 6213 | 2619 (42.2) | 2619 (42.2) | 369 (5.9) | 1421 (22.9) | 519 (8.4) |  |
| **Age (years)** |  | 51.2 (10.8) | 52.0 (10.6) | 52.0 (10.6) | 52.9 (9.2) | 55.8 (9.1) | 48.6 (10.3) |  |
| **Sex N (%)** | Female | 3158 (50.8) | 1264 (48.3) | 1264 (48.3) | 163 (44.2) | 831 (58.5) | 211 (40.7) |  |
| **Smoking N (%)** | Ever-Smoker | 2919 (47.0) | 1255 (47.9) | 1255 (47.9) | 195 (52.8) | 726 (51.1) | 235 (45.3) |  |
| **Charlson N (%)** | 0 | 2683 (43.2) | 1061 (40.5) | 1061 (40.5) | 147 (39.8) | 336 (23.6) | 283 (54.5) |  |
|  | 1 | 1990 (32.0) | 862 (32.9) | 862 (32.9) | 128 (34.7) | 574 (40.4) | 150 (28.9) |  |
|  | 2 | 1476 (23.8) | 667 (25.5) | 667 (25.5) | 89 (24.1) | 487 (34.3) | 86 (16.6) |  |
|  | 3 | 64 (1.0) | 29 (1.1) | 29 (1.1) | 5 (1.4) | 24 (1.7) | 0 (0.0) |  |
| **BMI** |  | 28.8 (6.1) | 29.1 (6.1) | 29.1 (6.1) | 28.8 (5.1) | 28.5 (5.9) | 29.4 (6.3) | 447 (7.1%) |
| **SBP (mmHg)** |  | 158.5 (25.8) | 162.5 (16.8) | 162.5 (16.8) | 131.1 (9.5) | 187.5 (19.6) | 151.1 (7.1) |  |
| **DBP (mmHg)** |  | 94.8 (12.7) | 96.9 (11.9) | 96.9 (11.9) | 84.1 (9.8) | 101.8 (12.3) | 95.5 (9.4) | 42 (0.6%) |
| **Cholesterol (mmol/L)** |  | 5.9 (1.6) | 6.0 (1.2) | 6.0 (1.2) | 5.5 (1.4) | 6.0 (1.3) | 5.8 (1.4) | 742(11.9%) |
| **27-year All-cause mortality N (%)** |  | 1435 (23.1) | 622 (23.7) | 622 (23.7) | 69 (18.7) | 521 (36.7) | 97 (18.7) |  |
| **10 year fatal/ non-fatal CV outcomes N (%)** |  | 1335 (21.5) | 612 (23.4) | 612 (23.4) | 67 (18.2) | 389 (27.4) | 91 (17.5) |  |
| **BP Classes Birmingham Model N (%)** | Normal/Target | 1645 (26.5) | 0 (0.0) | 0 (0.0) | 366 (99.2) | 0 (0.0) | 0 (0.0) |  |
|  | Normal/Target-WC | 2299 (37.0) | 1640 (62.6) | 1640 (62.6) | 0 (0.0) | 199 (14.0) | 460 (88.6) |  |
|  | Hypertension-Masked | 9 (0.1) | 0 (0.0) | 0 (0.0) | 3 (0.8) | 0 (0.0) | 0 (0.0) |  |
|  | Hypertension-WC | 2093 (33.7) | 862 (32.9) | 862 (32.9) | 0 (0.0) | 1215 (85.5) | 16 (3.1) |  |
|  | Hypertension | 167 (2.7) | 117 (4.5) | 117 (4.5) | 0 (0.0) | 7 (0.5) | 43 (8.3) |  |
| **BP Classes Gdańsk Model N (%)** | Normal/Target | 1267 (20.4) | 0 (0.0) | 0 (0.0) | 292 (79.1) | 0 (0.0) | 0 (0.0) |  |
|  | Normal/Target-WC | 1264 (20.3) | 800 (30.5) | 800 (30.5) | 0 (0.0) | 230 (16.2) | 234 (45.1) |  |
|  | Hypertension-Masked | 386 (6.2) | 0 (0.0) | 0 (0.0) | 77 (20.9) | 0 (0.0) | 0 (0.0) |  |
|  | Hypertension-WC | 2176 (35.0) | 981 (37.5) | 981 (37.5) | 0 (0.0) | 1162 (81.8) | 33 (6.4) |  |
|  | Hypertension | 1120 (18.0) | 838 (32.0) | 838 (32.0) | 0 (0.0) | 29 (2.0) | 252 (48.6) |  |

## Supplementary Table 7:

Hyperparameters for the machine learning models for the three cohorts. k-NN: k-nearest neighbours; MLR: multinomial logistic regression; SVM: support vector machine.

| Cohort | Glasgow | Poland | Gdańsk |
| --- | --- | --- | --- |
| XGBoost | max_depth = 9 min_child_weight = 1 colsample_bytree = 1 n_estimators = 47 learning_rate = 0.222 | max_depth = 3 min_child_weight = 1 colsample_bytree = 1 n_estimators = 17 learning_rate = 0.324 | max_depth = 8 min_child_weight = 27 colsample_bytree = 0.749 n_estimators = 97 learning_rate = 0.939 |
| k-NN | n_neighbors = 1 weights = 'uniform' | n_neighbors = 65 weights = 'distance' | n_neighbors = 6 weights = 'uniform' |
| Decision Tree | min_samples_split = 3 max_depth = 51 min_samples_leaf = 1 | min_samples_split = 64 max_depth = 4 min_samples_leaf = 51 | min_samples_split = 2 max_depth = 5 min_samples_leaf = 4 |
| Random Forest | n_estimators = 40 min_samples_split = 2 min_samples_leaf = 1 max_depth = 18 | n_estimators = 80 min_samples_split = 32 min_samples_leaf = 1 max_depth = 51 | n_estimators = 42 min_samples_split = 9 min_samples_leaf = 1 max_depth = 51 |
| Naive Bayes | Gaussian Naïve Bayes | Multinomial Naïve Bayes | Bernoulli Naïve Bayes |
| SVM | kernel='linear' | kernel='linear' | kernel='linear' |
| MLR | kernel='linear' | kernel='linear' | kernel='linear' |

**References**

1. Lip S, Tan LE, Jeemon P, McCallum L, Dominiczak AF, Padmanabhan S. Diastolic Blood Pressure J-Curve Phenomenon in a Tertiary-Care Hypertension Clinic. Hypertension 2019;**74**(4):767-775.

2. Berrar D. Cross-Validation. In: Ranganathan S, Gribskov M, Nakai K, Schönbach C, (eds). *Encyclopedia of Bioinformatics and Computational Biology*. Oxford: Academic Press; 2019, 542-545.

3. Hastie T, Tibshirani R, Friedman JH. *The elements of statistical learning*. New York: Springer 2009.

4. Huang C, Li SX, Caraballo C, Masoudi FA, Rumsfeld JS, Spertus JA*, et al.* Performance Metrics for the Comparative Analysis of Clinical Risk Prediction Models Employing Machine Learning. Circ Cardiovasc Qual Outcomes 2021;**14**(10):e007526.

5. Moons KG, Altman DG, Reitsma JB, Ioannidis JP, Macaskill P, Steyerberg EW*, et al.* Transparent Reporting of a multivariable prediction model for Individual Prognosis or Diagnosis (TRIPOD): explanation and elaboration. Ann Intern Med 2015;**162**(1):W1-73.

6. Pedregosa F, Varoquaux G, Gramfort A, Michel V, Thirion B, Grisel O*, et al.* Scikit-learn: Machine Learning in Python. Journal of Machine Learning Research 2011;**12**:2825-2830.

7. Parvandeh S, Yeh HW, Paulus MP, McKinney BA. Consensus features nested cross-validation. Bioinformatics 2020;**36**(10):3093-3098.

8. Hicks SA, Strumke I, Thambawita V, Hammou M, Riegler MA, Halvorsen P*, et al.* On evaluation metrics for medical applications of artificial intelligence. Sci Rep 2022;**12**(1):5979.
